# Altered Microglial Plasticity in the Periaqueductal Grey of Pre-Symptomatic *Mecp2*-Heterozygous Mice Following Early-Life Stress

**DOI:** 10.1007/s12017-025-08867-9

**Published:** 2025-06-17

**Authors:** Maria Abellán-Álvaro, Lidia Primo-Hernando, Elena Martínez-Rodríguez, Enrique Lanuza, Mónica Santos, Carmen Agustín-Pavón, Jose V. Torres-Pérez

**Affiliations:** 1 Department of Cellular Biology, Functional Biology and Physical Anthropology, University of Valencia, Burjassot, 46100 Valencia, Spain; 2 Unitat Predepartamental de Medicina, Universitat Jaume I, Castelló de La Plana, Spain; 3 Department of Biomedical Sciences, Faculty of Health Sciences, Institute of Biomedical Sciences, Cardenal Herrera-CEU University, CEU Universities, Valencia, Spain; 4 CNC-Centre for Neuroscience and Cell Biology, University of Coimbra, Coimbra, Portugal; 5 CIBB-Center for Innovative Biomedicine and Biotechnology, University of Coimbra, Coimbra, Portugal; 6 Institute for Interdisciplinary Research, University of Coimbra, Coimbra, Portugal

**Keywords:** Maternal separation, Rett syndrome, IBA1, Central grey, Fractal analysis, Cellular circularity

## Abstract

Rett syndrome (RTT), a severe neurodevelopmental disorder primarily affecting girls, is commonly caused by *MECP2* loss-of-function mutations. Key symptoms include motor impairments, typical hand stereotypies and intellectual disability. Moreover, although not thoroughly studied, anxiety, heightened stress sensitivity, and aberrant pain perception are also an important component of the RTT phenotype. Emerging evidence suggests that early-life stress (ELS) worsens *Mecp2*-related phenotypic alterations in mice. Microglia, the resident immune cells within the central nervous system, play a critical role in RTT pathophysiology, yet the combined impact of ELS and *Mecp2* deficiency on microglia has not been studied. Previously, we observed reduced activation of the periaqueductal grey (PAG, a cerebral structure involved in pain modulation, autonomic control, and defensive behaviours) in *Mecp2*-heterozygous (*Mecp2*-het) mice after thermal stimulation. Here, we investigated the impact of ELS on microglia morphology in the PAG under *Mecp2* deficiency. To this end, we analysed microglia in the PAG of presymptomatic *Mecp2*-het mice previously subjected to maternal separation (MS) as a model of ELS, alongside corresponding control animals. Brain sections were immunolabelled for IBA1, a pan-microglial marker. Microglial cells within the PAG were evaluated for expression levels, morphological characteristics, and fractal properties. While global PAG analyses showed minimal differences, subdivision-specific analyses revealed significant microglial alterations. These findings suggest that ELS exacerbates *Mecp2*-related neurodevelopmental deficits, impairing microglia in a region-specific manner. Our data points to a microglial failure to morphologically adapt, rather than overt structural loss, in the PAG that may underlie some of the neurological dysfunctions observed in RTT.

**Supplementary Information:**

The online version contains supplementary material available at 10.1007/s12017-025-08867-9.

## Background

Rett syndrome (RTT) is a neurodevelopmental disorder primarily affecting girls. It is characterised by a period of apparently normal development followed by a progressive decline (Amir et al., 1999). Anxiety and aberrant pain perception are also prominent features (Barnes et al., 2015; Barney et al., 2015; Downs et al., 2010). RTT’s primary cause is de novo loss-of-function mutations in the methyl-CpG binding protein 2 gene (*MECP2*), on chromosome Xq28. MeCP2 is a transcriptional modulator with a pivotal role in neuronal maturation, synaptic plasticity, and activity- and context-dependent gene expression (Irfan et al., 2022; Johnston et al., 2001; Lee et al., 2021; Sharifi & Yasui, 2021).

Emerging evidence suggests that early-life stress (ELS) can exacerbate the phenotypic alterations associated with MeCP2 deficiency (Abellán-Álvaro et al., 2021; Torres-Pérez et al., 2022). In juvenile *Mecp2*-heterozygous female mice (*Mecp2*-het), maternal separation (MS, an ELS model), induces persistent modifications that lead to reduced anxiety-related behaviours (Abellán-Álvaro et al., 2021). Therefore, MeCP2 seems to interplay with environmental adversity. MeCP2 is also linked to stress vulnerability, with altered expression after ELS (Murgatroyd et al., 2009) and associations with PTSD severity (Cosentino et al., 2023).

Microglia, the resident macrophages in the central nervous system (CNS), are critical in RTT pathophysiology (Derecki et al., 2012; Wang, 2012). Microglial morphology reflects their function, with a ramified shape for immune-surveillance and an amoeboid form during activation, linked to phagocytosis, cytokine release, and motility (Ito et al., 1998; Ohsawa et al., 2004; Vidal-Itriago et al., 2022). This morphological plasticity can be assessed using immunofluorescence for ionised calcium-binding adaptor molecule 1 (IBA1) (Imai et al., 1996; Ito et al., 1998; Young & Morrison, 2018).

Microglia can express MeCP2 (Cronk et al., 2015; Schafer et al., 2016). Additionally, microglia could contribute to RTT via other cell types rendered vulnerable by the lack of MeCP2 (Maezawa & Jin, 2010; Schafer et al., 2016). Importantly, ELS is known to exert profound and lasting effects on microglia influencing neurodevelopment (Smail & Lenz, 2024), which might increase susceptibility to neuropsychiatric conditions (Young & Morrison, 2018). Nonetheless, the cumulative effect of ELS and MeCP2-defficiency on microglia remains largely unexplored.

The periaqueductal grey (PAG) has been implicated in RTT neurophysiology (Belichenko et al., 2008; Glaze, 2005). It is anatomically divided into four columnar subdivisions with distinct roles: the dorsomedial (dmPAG) and dorsolateral (dlPAG) mediate active defensive responses (e.g., escape), while the ventrolateral (vlPAG) in passive responses (e.g., freezing and analgesia); and the lateral PAG (lPAG) has an integrative role (Coulombe et al., 2016; La-Vu et al., 2022; Zhang et al., 2024). Despite its role, little is known about how ELS affects microglia in the PAG.

We have recently demonstrated that the PAG of *Mecp2*-het mice subjected to thermal stimulation exhibit reduced activation which associates with decreased levels of cannabinoid receptor 1 (CB1) within the PAG (Cuitavi et al., 2025). Additionally, vlPAG dysfunction has been previously associated with reduced opioid analgesic efficacy, potentially driven by microglia (Doyle et al., 2017).

Together, these findings support the hypothesis that ELS and *Mecp2* deficiency might exert a cumulative effect on microglial developmental trajectories within the PAG. Given MeCP2’s broader role in stress-related pathophysiology, ELS could exacerbate microglial impairments in *Mecp2*-heterozygous mice, contributing to altered stress responsiveness.

## Material and Methods

### Animals

The brain sections used here were obtained from spare brain series collected from animals (*n* = 19) included in a previous publication (Abellán-Álvaro et al., 2021). Briefly, both *Mecp2*-het females and their wild-type (WT) littermates originated from our colony (Jackson Laboratory; stock #003890, B6.129P2(C)-*Mecp2*^*tm1.1Bird/J*^) (Guy et al., 2001). Breeding was achieved by crossing *Mecp2*-het females with C57Bl/6 J WT males. Experimental females were weaned at postnatal day (PND) 23 and housed in groups of 2–5 animals in standard laboratory cages under controlled conditions, including a temperature of 22 °C, controlled humidity, an inverted 12:12-h light/dark cycle, and ad libitum access to food and water. Genotyping (following Jackson Laboratory’s protocol) was carried out using DNA extracted from earplug biopsies. Behavioural testing was conducted during the dark phase of the light/dark cycle to align with the animals' natural activity period.

All the procedures were carried out in accordance with the EU directive 2010/63/EU and protocols approved by the Ethics in Animal Experimentation Committee of the University of Valencia.

We focused on presymptomatic females to examine early ELS–*Mecp2* interactions before motor symptom onset, in line with human evidence linking reduced MeCP2 levels to stress vulnerability in trauma-exposed females (Cosentino et al., 2023).

### Early-Life Stress (ELS)

To induce early-life stress (ELS), the maternal separation (MS) protocol was implemented as previously described in a prior publication (Abellán-Álvaro et al., 2021). Briefly, from PND 3 to PND 21, pups (WT-MS and *Mecp2*-het-MS) were separated from their dams and placed in a new cage (all litter pups as a group) with sawdust and a warming red light for 3 h daily before being returned to their home cage. Control animals remained undisturbed (standard care, SC) with their dams in the home cage until weaning (WT-SC, *Mecp2*-het-SC). At 6 weeks of age, all animals underwent a battery of behavioural tests (further details in previous publication).

### Histology

One hour after last behavioural test, six-week-old animals were anaesthetised with ketamine (75 mg/Kg) and medetomidine (1 mg/Kg) and perfused with saline followed by 4% paraformaldehyde. Brains were post-fixed, cryoprotected in 30% sucrose, frozen, and sectioned coronally into 40-μm slices. Sections were stored in 30% sucrose with 0.02% sodium azide until use. Spare brain series (one out of six) from a subset of animals were selected for this study (WT-SC, *n* = 4; *Mecp2*-het-SC, *n* = 5; WT-MS, *n* = 6; *Mecp2*-het-MS, *n *= 4).

### IBA1 Immunofluorescence

One brain series from each animal was processed for immunofluorescence against IBA1. IBA1 (ionised calcium-binding adaptor molecule 1) is a widely used pan-microglial marker that labels both resting and activated microglia commonly used to assess changes in microglial presence/morphology, including potential immune activation within CNS (Imai et al., 1996; Ito et al., 1998; Young & Morrison, 2018). Brain slices were first incubated in 1% sodium borohydride in 0.05 M TBS for 30 min at room temperature (RT) to block endogenous tissue fluorescence. The sections were then placed in a blocking solution containing 4% normal donkey serum (NDS) in TBS-Tx 0.3% for 1 h at RT. Subsequently, sections were incubated for 72 h at 4 °C with the primary antibody goat anti-IBA1 (1:400, AB5076, Abcam) diluted in TBS-Tx 0.3% with 2% NDS. Following primary antibody incubation, slices were treated for 90 min at RT with the secondary antibody Alexa Fluor 488-conjugated donkey anti-goat IgG (1:200, A-11055, Invitrogen) diluted in TBS-Tx 0.3% with 2% NDS. Finally, sections were stained with DAPI (4’,6’-diamino-2-feniindol, 1:100, in TBS for 5 min), mounted onto gelatinised slides and cover-slipped using fluorescence mounting medium (FluorSave Tm Reagent; Dako, Glostrup, Denmark).

### Image Acquisition and Processing

IBA1 immunofluorescent images from PAG’s four major anatomical subdivisions, dmPAG, dlPAG, lPAG, and vlPAG (Paxinos & Franklin, 2004), were taken at the section most central to the Bregma levels -4.10 to -5 (one section per animal). When present, both left and right hemisphere images were taken from the dlPAG and lPAG. PAG images were taken from both *Mecp2*-het mice and WT siblings. Image acquisition was conducted using z-stacks (around 7 planes) using an Olympus FV1000 confocal microscope via the Olympus Fluoview Ver.4.2 software at 20 × magnification. Image acquisition and analysis were conducted under blinded conditions regarding genotype and treatment.

#### Percentage of IBA1-ir Area

BioFormats plugin from ImageJ (https://imagej.net/formats/bio-formats) was used to convert the “.oif” files generated by the confocal microscope software into “.tif” files. Resulting images were auto-thresholded using Shanbhang’s method to calculate percentage of IBA1-immunoreactivity (IBA1-ir) area within each image. If confocal images showed a portion outside neuropil (the aqueduct), total area was corrected to exclude those portions. Data obtained on the percentage of IBA1-ir can be found within supplementary material (Supplementary Table 1).

#### Morphometric Analysis of Microglial Cells

For the morphometric assessment, we performed Skeleton analysis using ImageJ software with the AnalyzeSkeleton (2D/3D) plugins (https://imagej.net/plugins/analyze-skeleton/; (Arganda-Carreras et al., 2010)) similarly as previously described by Young and Morrison (Young & Morrison, 2018). This morphometric analysis was done to determine the activation state of the microglia. Briefly, fluorescence images were converted to 8-bit grayscale, and brightness and contrast adjusted to optimise the visibility of microglial processes without altering pixel values. An Unsharp Mask filter (default settings: pixel radius 3, mask weight 0.6) was then applied to enhance edge features, and the despeckle function was used to remove salt-and-pepper noise. Images were converted to binary (thresholding) and further refined (despeckle, closing gaps -up to 2 pixels-, and removing outliers). Resulting processed binary images were skeletonised using the “Skeletonize” function, and skeleton analysis conducted with the AnalyzeSkeleton (2D/3D) plugin. Results from the “Branch Information” output, including number of branches, end-point voxels, average branch length and maximum branch length, were exported to Excel for further analysis (data can be found in Supplementary Table 2). Although modest in magnitude, even small changes in these parameters (e.g., end-point voxels) can reflect meaningful shifts in microglial function (Dayananda et al., 2023).

#### Fractal Analysis of Microglial Cells

Fractal analysis was performed using the FracLac plugin (https://imagej.nih.gov/ij/plugins/fraclac/fraclac.html). As with the previous analyses, the procedures described by Young and Morrison (Young & Morrison, 2018) were followed with small variation (stated below). This method provided less subjective information regarding microglial morphology, offering quantitative measurements to study continuous cellular transformations.

From the binarized images generated in the previous step (see “Morphometric analysis of microglial cells“) we located the first IBA1-ir positive cells in the upper-right corner of the images which had completed branching and nuclei (DAPI) as our region of interest (ROI) for that image. Same ROI-size was used in all images. Next, cells were isolated in the resulting image by removing adjacent cell processes and connecting fragmented processes with the paintbrush tool. Cells were converted into an outline and scanned with the FracLac plugin with the following conditions: box counting (BC) selected, and grid orientations set to 4. Results, containing density, span ratio, circularity, fractal dimension, and lacunarity were also exported to Excel for further analysis (data can be found in Supplementary Table 3).

### Statistical Analysis

The PAG was first analysed globally (average value combining data from all main subdivision) and then divided in its major anatomical subdivisions: dmPAG, dlPAG, lPAG, and vlPAG. For subdivisions with bilateral representations (dlPAG and lPAG), data were averaged across both hemispheres, whenever present.

All statistical analyses were performed in R (Version: 2024.12.1 + 563), while data visualization and graphs were generated using GraphPad Prism 9.0.2 for Windows (GraphPad Software, San Diego, California, USA). Normality and homoscedasticity were assessed using the Shapiro–Wilk test (shapiro.test() from the stats package) and Levene’s test (leveneTest() from the car package), respectively. For normally distributed data with equal variances, a two-way ANOVA was conducted using the aov() function from the stats package. The model included treatment (SC vs. MS) and genotype (WT vs *Mecp2*-het) as between-subject factors. Post hoc analyses were performed using Bonferroni-corrected pairwise t-tests (pairwise.t.test() from the stats package, with p.adjust.method = ”bonferroni”). For non-normally distributed data, an Aligned Rank Transformation (ART) ANOVA was conducted using the art() function from the ARTool package. Post hoc analyses for significant main effects and interactions were performed using art.con() from the same package, followed by Bonferroni-corrected pairwise comparisons (pairwise.t.test() from the stats package, with p.adjust.method = ”bonferroni”). Statistical significance was set at *p* < 0.05 for all analyses. Full coded scripts can be found within supplementary materials.

## Results

For all the analyses performed, the PAG was first evaluated globally by averaging data from all major subdivisions. This global analysis compared treatment conditions (SC vs MS) and genotypes (WT vs *Mecp2*-het) as between-subject factors. Subsequently, the same analyses were performed separately for the four primary anatomical and functional subdivisions of the PAG to identify potential differences among them. For dlPAG, lPAG and vlPAG, data from the left and right hemispheres were averaged when both sides were present.

### ELS impacts IBA1-ir Area at the vlPAG Exclusively

**Fig. 1:**
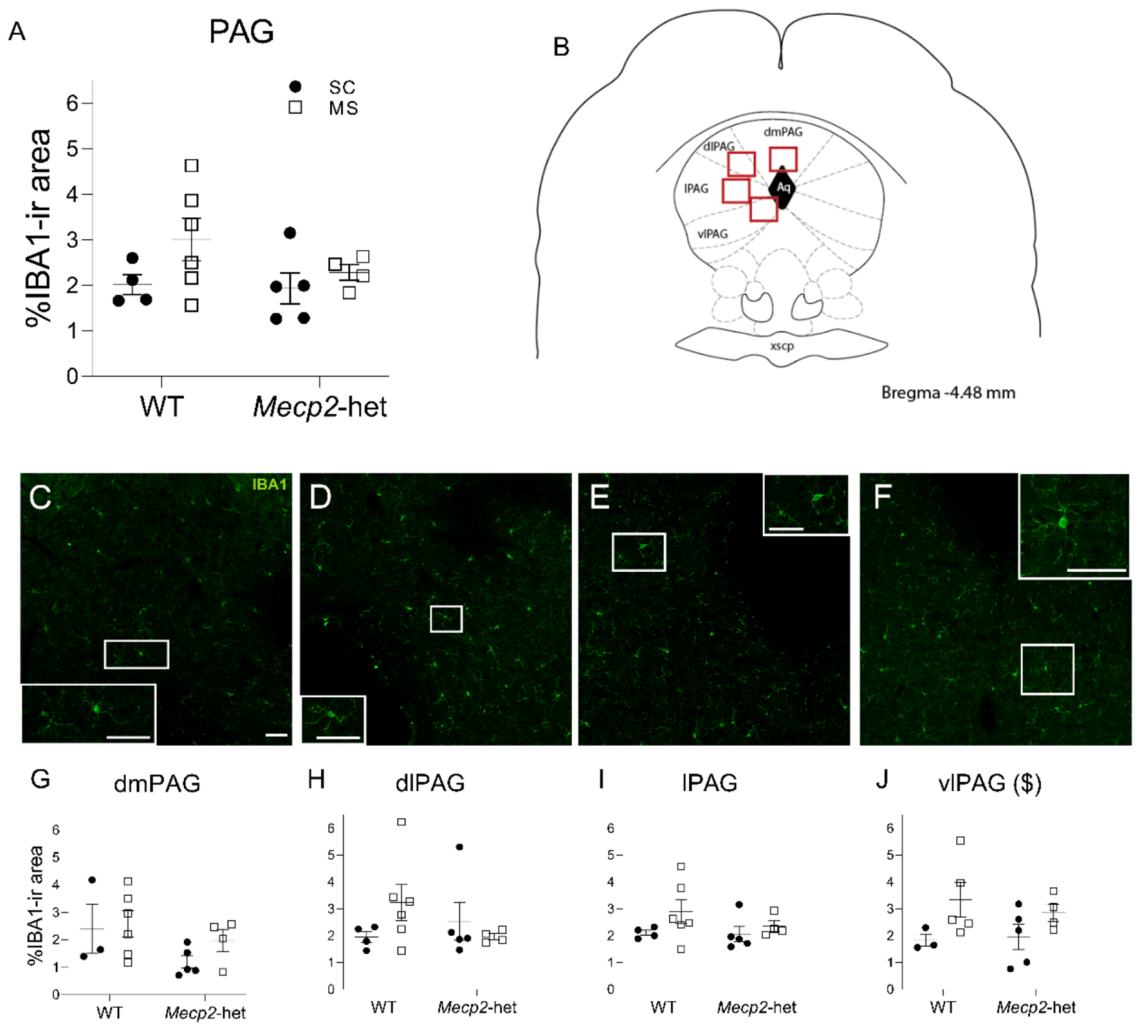
Percentage of IBA1-immunoreactive area in the PAG. **A** Percentage of area IBA1-ir considering the PAG (average values combining the four main subdivisions). **B** Stereotaxic representation of a coronal section of the PAG at Bregma -4.6, according to (Paxinos & Franklin, 2004); squared regions delineate the areas studied according to the four main subdivisions: dosomedial PAG (dmPAG; **C** and **G**), dorsolateral PAG (dlPAG; **D** and **H**), lateral PAG (lPAG; **E** and I), and ventrolateral PAG (vlPAG; **F** and **J**). **C**–**F** representative images of the four main subdivisions depicting IBA1 (green) and DAPI (blue); all images at same magnification; scale bars represent 50 µm. A negative control (section processed without primary antibody) confirming the specificity of IBA1 staining is shown in Supplementary Fig. 1. **G–J** Percentage of area IBA1-ir for the four main subdivisions. Graphs show individual values (black circles: animals subjected to standard care -SC; white squares: animals subjected to maternal separation -MS) and mean ± standard error mean (SEM) from both wildtype (WT) and *Mecp2*-heterozygous (*Mecp2*-het) animals. ($) significant effect for the factor ‘treatment’

The percentage of IBA1-ir area (Fig. 1) was measured as a straightforward and rapid method to evaluate broad differences in the extent of microglial coverage within the neuropil, serving as an indirect measure of their overall functional state. The statistical analysis on the PAG as a whole (combining all major subdivisions) revealed no significant differences for the factors ‘treatment’ and ‘genotype’, or their interaction (ANOVA; treatment: F(1,13) = 1.757, *p* = 0.208; genotype: F(1,13) = 1.743, *p* = 0.209; treatment*genotype: F(1,13) = 0.287, *p* = 0.601). Similarly, no significant differences in IBA1-ir area were observed across the dmPAG, dlPAG, and lPAG subdivisions (ART; *p* > 0.05). In opposition, statistical analysis revealed a significant effect of ‘treatment’ at the vlPAG (ANOVA; F(1,13) = 5.314, *p* = 0.0383), while ‘genotype’ (F(1,13) = 0.697, *p* = 0.4188) and their interaction (F(1,13) = 0.343, *p* = 0.5682) remained non-significant. However, post hoc pairwise comparisons did not reveal any significant difference.

### Region-Specific Microglial Morphological Alterations in the PAG of Mecp2-het

Morphological analysis (skeleton analysis) was processed similarly as in Young and Morrison (Young & Morrison, 2018) and included average number of branches per cell, average number of endpoints per cell, average branches (processes) length per cell and maximum branch length per cell.

**Fig. 2:**
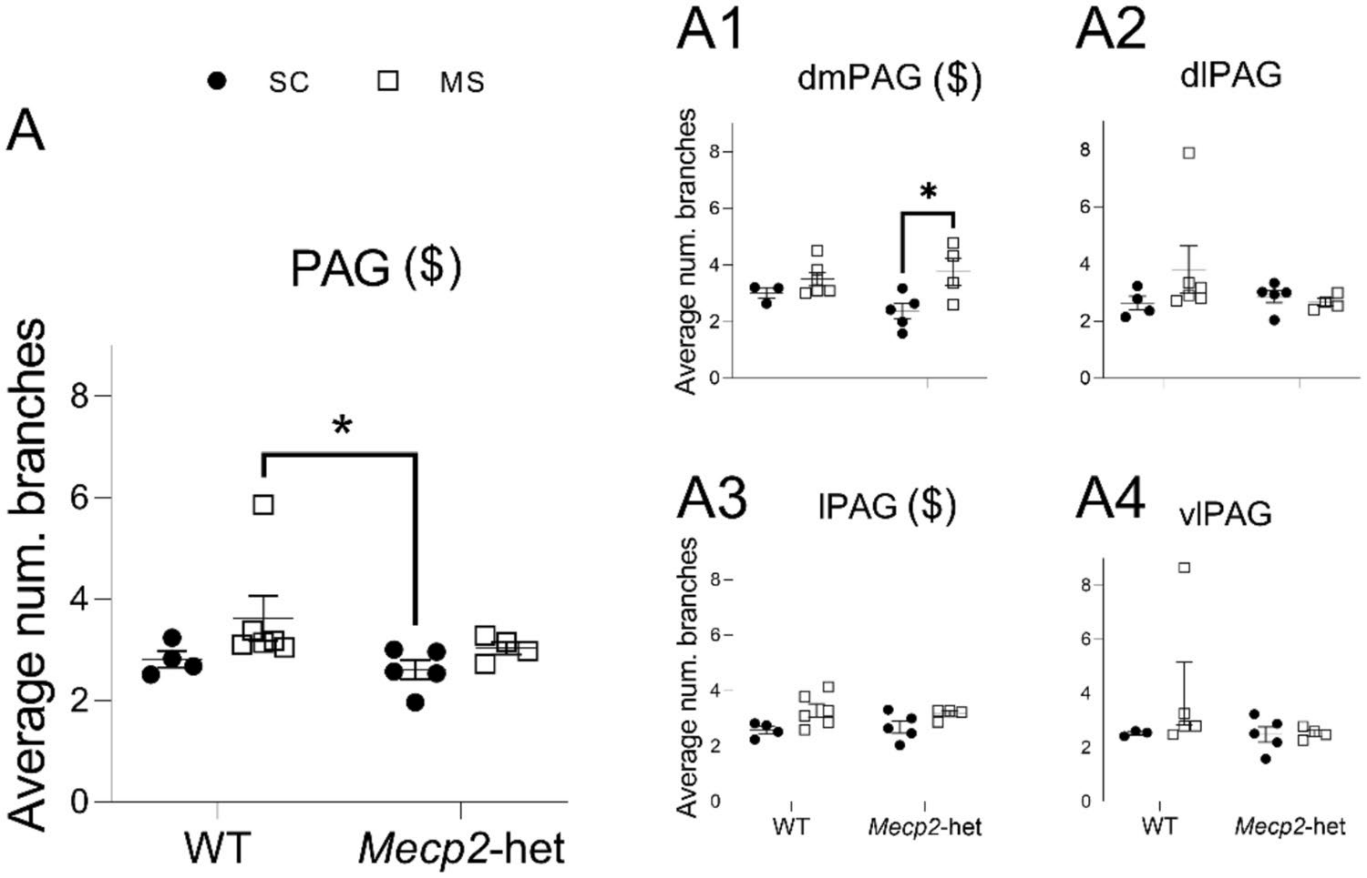
Average number of branches per microglial cells in the PAG. **A** Considering the PAG as a whole. Similar data from the four major subdivisions are presented as: **A1** dmPAG, **A2** dlPAG, **A3** lPAG, and **A4** vlPAG. Graphs show individual values (black circles: animals subjected to standard care -SC; white squares: animals subjected to maternal separation -MS) and mean ± SEM from both wildtype (WT) and *Mecp2*-heterozygous (*Mecp2*-het) animals. ($) significant effect for the factor ‘treatment’. *p < 0.05 *vs* corresponding pairwise comparison

Our analysis of the average number of microglial branches per cell considering the PAG as a whole (Fig. 2) revealed a significant effect of ‘treatment’ (ART; F(1,15) = 10.863, *p* = 0.0049) while ‘genotype’ (F(1,15) = 2.45, *p* = 0.138) and the interaction treatment*genotype (F(1,15) = 0.17, *p* = 0.688) were not significant. Although post hoc pairwise comparisons revealed a significant difference between WT-MS vs *Mecp2*-het-SC (*p* = 0.017), no other comparison was significant. When examining each PAG subdivision separately, we identified a significant effect of ‘treatment’ specifically in the dmPAG (ANOVA; F(1,14) = 8.903, *p* = 0.0099; Fig. 2A1) and in the lPAG (ANOVA; F(1,14) = 5.969, *p* = 0.0284; Fig. 2A3). Notably, post hoc analysis showed a significant difference in the dmPAG of *Mecp2*-het; specifically, microglial cells of *Mecp2*-het mice subjected to MS exhibited a significantly higher number of branches compared to those kept under SC (*p* = 0.044). No other comparisons reached statistical significance for this morphological parameter (*p* > 0.05).

**Fig. 3:**
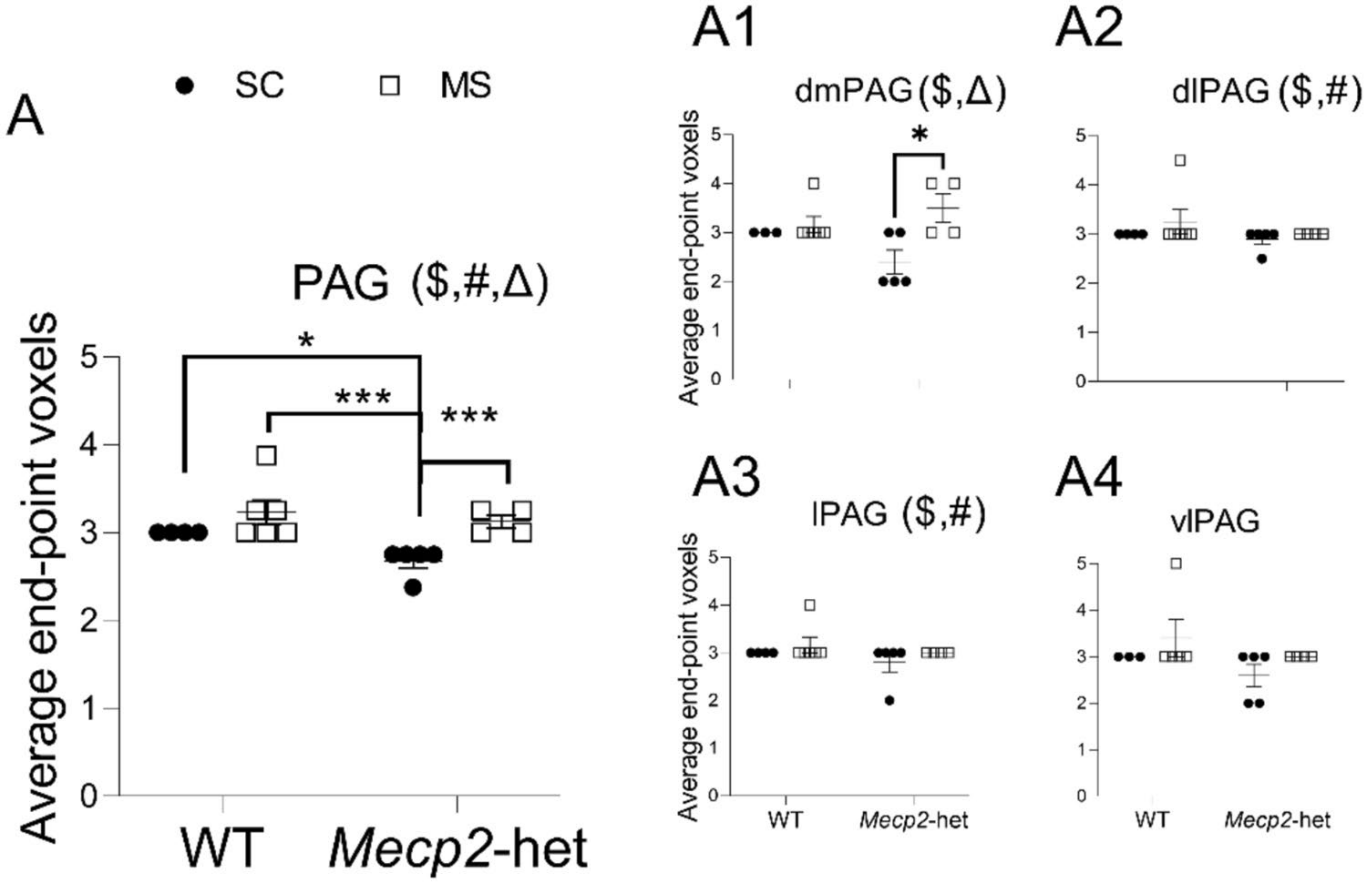
Average end-point voxels per microglial cells in the PAG. **A** Considering the PAG as a whole. Similar data from the four major subdivisions are presented as: **A1** dmPAG, **A2** dlPAG, **A3** lPAG, and **A4** vlPAG. Graphs show individual values (black circles: animals subjected to SC; white squares: animals subjected to MS) and mean ± SEM from both WT and *Mecp2*-het animals. (Δ) significant effect for the interaction treatment*genotype; ($) significant effect for the factor ‘treatment’; (#) significant effect for the factor ‘genotype’. *p < 0.05, ***p < 0.001 *vs* corresponding pairwise comparison

The analysis of the average number of endpoints per microglial cell revealed a significant effect of ‘treatment’ (ART; F(1,15) = 62.86, p = 9.61·10^–7^; Fig. 3), ‘genotype’ (F(1,15) = 20.67, *p* = 0.00039) and their interaction (F(1,15) = 5.49, *p* = 0.033) in the PAG, as a whole. Post hoc pairwise comparison showed this to be driven by an increase in the *Mecp2*-het subjected to MS when compared to those under SC (*p* = 0.00071). Interestingly, there was also a difference between WT and *Mecp2*-hets under SC by which the latest showed a reduction in endpoint voxels (*p* = 0.019) and between WT-MS and *Mecp2*-het-SC (*p* = 0.00016).

Assessing the average number of endpoints at the different subdivisions also showed significant differences. At the dmPAG, there was an effect of ‘treatment’ (ART; F(1,14) = 7.05, *p* = 0.019; Fig. 3A1) and the interaction treatment*genotype (F(1,14) = 5.17, *p* = 0.039), while ‘genotype’ alone did not reach significance (F(1,14) = 0.73, *p* = 0.41). At both the dlPAG (Fig. 3A2) and lPAG (Fig. 3A3), there was an effect of ‘genotype’ (ART; dlPAG: F(1,15) = 13.37, *p* = 0.0023; lPAG: F(1,14) = 11.17, *p* = 0.0049)) and ‘treatment’ (dlPAG: F(1,15) = 13.37, *p* = 0.0023; lPAG (F(1,14) = 11.10, *p* = 0.0049)) while their interaction was not significant (dlPAG: F(1.15) = 0.06, *p* = 0.82; lPAG: F(1,14) = 0.11, *p* = 0.75). Post hoc analysis only revealed a significant pairwise comparison in the dmPAG by which MS resulted in an increase in endpoints in the *Mecp2*-hets (p = 0.02). No other significant differences where observed for this morphological measure (p > 0.05).

**Fig. 4:**
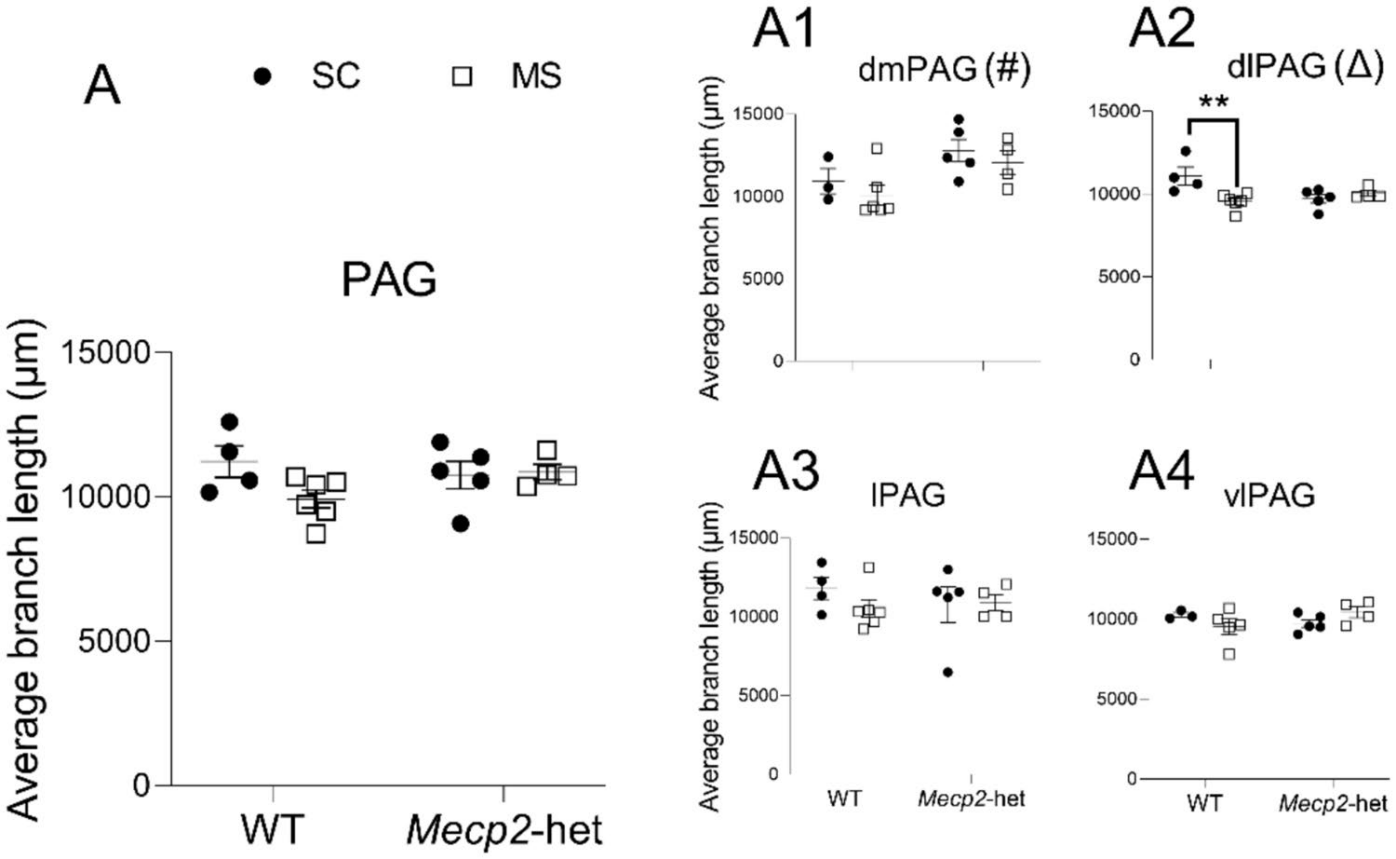
Average branch length per microglial cells in the PAG. **A** Considering the PAG as a whole. Similar data from the four major subdivisions are presented as: **A1** dmPAG, **A2** dlPAG, **A3** lPAG, and **A4** vlPAG. Graphs show individual values (black circles: animals subjected to SC; white squares: animals subjected to MS) and mean ± SEM from both WT and *Mecp2*-het animals. (Δ) significant effect for the interaction treatment*genotype; (#) significant effect for the factor ‘genotype’. **p < 0.01 *vs* corresponding pairwise comparison

We also examined the average branch length per microglial cell (Figs. 4). When assessing the PAG (all subareas averaged), no significant effects were found for either ‘treatment’ (ANOVA; F(1,15) = 2.277, *p* = 0.152) or ‘genotype’ (F(1,15) = 0.776, *p* = 0.392), nor for the interaction between these factors (F(1,15) = 2.842, *p* = 0.113; Fig. 4A). However, a significant effect of ‘genotype’ was observed in the dmPAG (ANOVA; F(1,14) = 9.305, *p* = 0.009; Figs. 4A1), although this did not result in significant pairwise differences (*p* > 0.05). Interestingly, a significant interaction treatment*genotype was detected for this parameter in the dlPAG (ART; F(1,15) = 9.008, *p* = 0.0089; Figs. 4A2), where post hoc analyses revealed significantly shorter branches in WT animals due to the earlier exposure to MS (*p* = 0.0077). Interestingly, there was a nearly significant difference between HET-SC and WT-SC (*p* = 0.052).

Finally, we assessed the maximum branch length per microglial cell as an additional morphological measure to evaluate microglial complexity (Supplementary Fig. 2). This analysis did not reveal any statistically significant effects for either ‘treatment’, ‘genotype’, or their interaction at any area (*p > *0.05).

### Fractal Analysis also Reveals Region-Specific Microglial Alterations in the PAG

We also used fractal analysis to assess microglial morphology (representative images at Supplementary Fig. 3) due to its greater precision than traditional morphometric methods to capture the intricate complexity and dynamic branching patterns characteristic of microglia (Young & Morrison, 2018). Fractal analysis relies on objective, algorithm-driven calculations, minimising subjective interpretation and inter-observer variability. Specifically, we have assessed the following parameters: circularity, lacunarity (*λ*), density, span ratio, and fractal dimension (D_β_), which reflect the geometric complexity and spatial distribution of microglial processes.

**Fig. 5:**
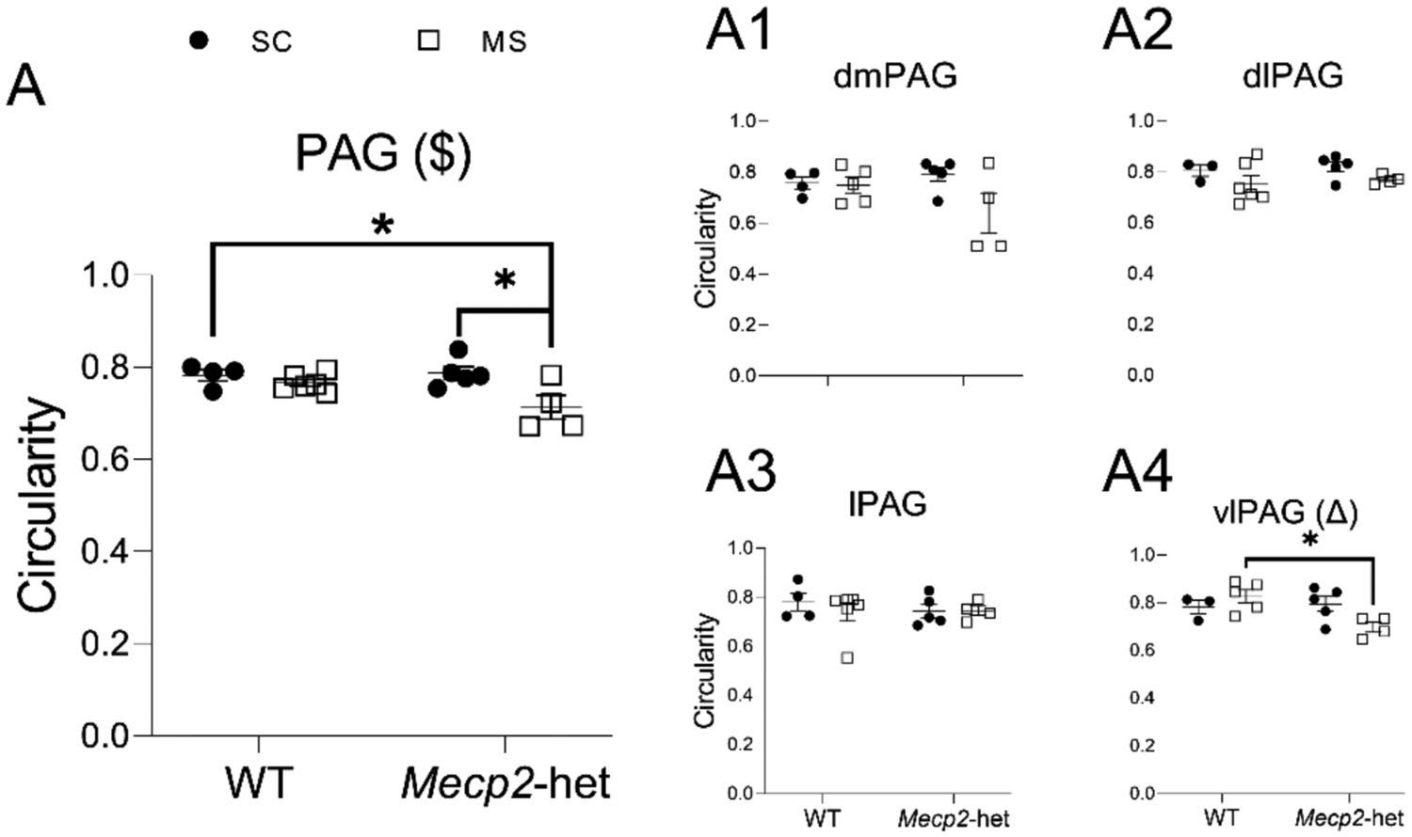
Circularity of PAG microglia (fractal analysis). **A** Considering the PAG as a whole. Similar data from the four major subdivisions are presented as: **A1** dmPAG, **A2** dlPAG, **A3** lPAG, and **A4** vlPAG. Graphs show individual values (black circles: animals subjected to SC; white squares: animals subjected to MS) and mean ± SEM from both WT and *Mecp2*-het animals. (Δ) significant effect for the interaction treatment*genotype; ($) significant effect for the factor ‘treatment’. *p < 0.05 *vs* corresponding pairwise comparison

Circularity serves to measure how closely a structure resembles a perfect circle. In microglial morphology, higher circularity (values closer to 1) indicates a more compact, rounded structure typically associated with reactive microglia, whereas lower circularity (values closer to 0) suggests a more complex, branched morphology characteristic of resting microglia. Our analysis revealed a significant effect of ‘treatment’ in the PAG as a whole (ANOVA; F(1,15) = 8.787, *p* = 0.00965; Fig. 5A), while neither ‘genotype’ (F(1,15) = 1.505, *p* = 0.239) nor the interaction treatment*genotype reached statistical significance (F(1,15) = 3.797, *p* = 0.0703). Post hoc comparisons identified a significant difference between *Mecp2*-het animals reared under SC and those mutants subjected to MS (*p* = 0.021), with the latter displaying significantly reduced circularity indicative of a more ramified microglial morphology following ELS. There was also a significant difference between WTs kept under SC and *Mecp2*-hets subjected to MS (*p* = 0.049).

When analysing the main PAG subdivisions, we found a significant effect of the interaction treatment*genotype in the vlPAG (ANOVA; F(1,13) = 5.816, *p* = 0.0314; Fig. 5A4), which post hoc analysis showed to be driven by a reduction in circularity in *Mecp2*-het mice exposed to MS when compared to WT animals subjected to the same ELS (*p* = 0.038), thus suggesting differential microglial responses to stress depending on genotype. No other subdivisions exhibited significant differences (*p* > 0.05; Fig. 5A1–A3).

**Fig. 6:**
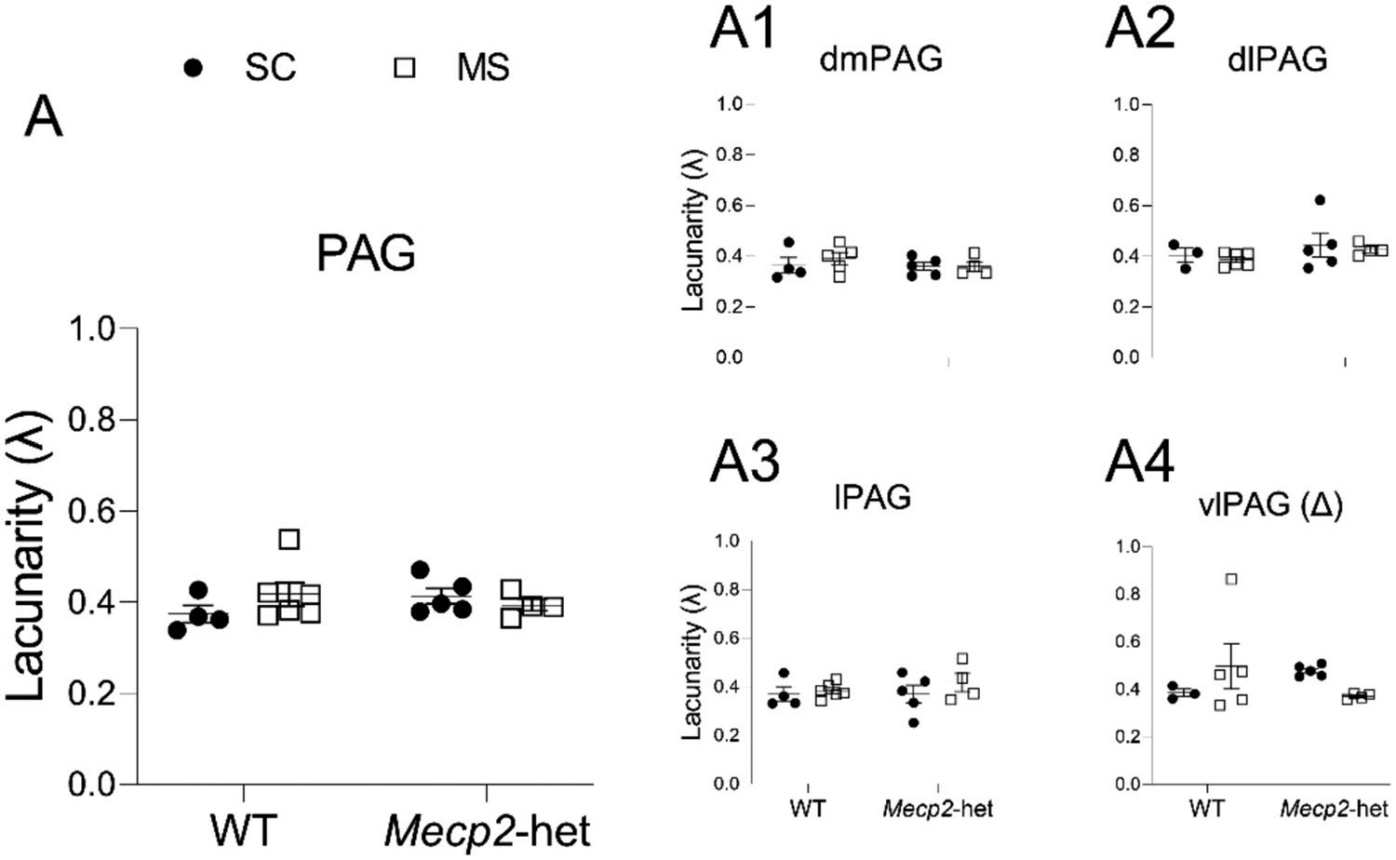
Lacunarity (λ) of PAG microglia (fractal analysis). **A** Considering the PAG as a whole. Similar data from the four major subdivisions are presented as: **A1** dmPAG, **A2** dlPAG, **A3** lPAG, and **A4** vlPAG. Graphs show individual values (black circles: animals subjected to SC; white squares: animals subjected to MS) and mean ± SEM from both WT and *Mecp2*-het animals. (Δ) significant effect for the interaction treatment*genotype

The analysis of lacunarity (Λ; Fig. 6), which measures the heterogeneity and texture of cellular architecture by assessing the distribution of gaps within the structure, did not reveal significant effects across the PAG (ART; F(1,15) = 0.023, *p* = 0.882). However, a significant interaction genotype*treatment was found in the vlPAG (ART; F(1,13) = 4.773, *p* = 0.048) suggesting that the spatial distribution of microglial processes in this subregion might be differentially affected by ELS depending on genotype. However post hoc analysis revealed no significant pairwise differences.

In contrast, the statistical analysis of microglial density, span ratio and fractal dimension (D_β_), in the PAG revealed no significant effects of ‘treatment,’ ‘genotype,’ or their interaction when considering the PAG as a whole or its subdivisions (*p* > 0.05). Cellular density (Supplementary Fig. 4A–A4), a measure of microglial distribution, showed no significant differences across groups. Similarly, the span ratio (Supplementary Figs. 4B–B4), which quantifies microglial spatial spread, did not exhibit significant effects at the global or regional level. Moreover, fractal dimension (D_β_; Supplementary Figs. 4C–C4), a measure of structural complexity, did not differ significantly across experimental conditions.

## Discussion

In the present study, we investigated the impact of ELS on PAG microglia, focusing on presymptomatic (6-week-old) *Mecp2*-het mice exposed to MS. While previous studies have characterised microglial alterations in *Mecp2*-deficient models, this is, to the best of our knowledge, the first research examining these effects within the PAG, a critical hub for pain modulation, defensive behaviours, and autonomic regulation. We assessed microglial characteristics through expression levels, morphological analysis, and fractal quantification. When considering the PAG, without distinguishing between its main functional–anatomical subdivisions, we identified differences solely in the number of microglial branches and endpoint voxels. Nonetheless, when analysing the functional–anatomical subdivisions separately, we observed a greater number of altered parameters than when assessing the PAG as a whole.

Crucially, we identified that WT mice subjected to ELS exhibited significantly reduced average branch length in the dlPAG, an effect that was not present in *Mecp2*-het mice, suggesting that wild-type microglia actively remodel in response to stress (Catale et al., 2020), whereas *Mecp2*-deficient microglia fail to exhibit such plastic changes. Additionally, genotype-dependent differences were found in other PAG subdivisions independent of ELS, reinforcing the idea that microglial alterations in *Mecp2*-deficient mice are region-specific. These findings highlight a more complex pattern of microglial dysregulation in RTT models than previously appreciated, particularly within a key brainstem region essential for pain and stress responses.

### Microglial from Both dmPAG and dlPAG Show Impaired Adaptation to ELS in Mecp2-het Mice

In the dmPAG, critical for mediating active defensive behaviours (Zhang et al., 2024), *Mecp2*-het mice subjected to MS exhibited a significant increase in the number of microglial branches and endpoints compared to SC counterparts from the same genotype, while treatment did not have this effect in WT’s microglia (Figs. 2A1 and 3A1). Importantly, the same cohort of animals used here was previously shown to exhibit altered hypothalamic–pituitary–adrenal (HPA) axis activity, with *Mecp2*-het mice displaying reduced activation of CRH-positive neurons in the PVN in response to testing in the elevated plus maze (Abellán-Álvaro et al., 2021), supporting the notion that stress responsiveness differs across genotypes.

Increased branching can be associated with enhanced immune-surveillance (Vidal-Itriago et al., 2022) or, alternatively, with a compensatory response characteristic of chronic conditions (Harry & Kraft, 2008), thus indicating an attempt by microglia to counterbalance disrupted neural circuitry. However, the absence of significant changes in other parameters, including branch length and fractal analysis, suggests that this plasticity may be structurally superficial, lacking functional integration with neural circuits. This partially aligns with previous research assessing microglia following ELS (Smail & Lenz, 2024).

In the dlPAG, also mediating active defensive behaviours (Coulombe et al., 2016; La-Vu et al., 2022; Zhang et al., 2024), ELS significantly decreased branch length specifically in WT mice (Fig. 4A2). This suggests that microglia in this region adopt a more reactive or less ramified morphology in response to early adversity (Catale et al., 2020). This dlPAG microglial retraction could reflect a heightened state of surveillance or a pro-inflammatory shift. Similar microglial morphological changes have been reported in other stress-responsive brain regions, such as the prefrontal cortex and amygdala, where ELS leads to increased microglial activation and altered synaptic interactions (Bolton et al., 2022)​. A reduction in branch length may therefore suggest a loss of microglial plasticity, potentially disrupting the homeostatic regulation of neuronal circuits involved in adaptive stress responses.

Interestingly, this effect is specific to WT animals as *Mecp2*-het mice did not display this stress-induced plasticity. This suggests that WT microglia are more responsive to environmental stressors, whereas *Mecp2*-deficient microglia either fail to undergo normal stress-induced remodelling or are already in a dysregulated state. Thus, similarly to what we observed in the dmPAG, dlPAG microlgia shows an impaired capacity to adapt to stress in *Mecp2*-hets, consistent with the potentially reduced microglial plasticity. This failure of microglia to appropriately remodel in response to environmental stress may contribute to the dysregulation of pain-related responses mediated by the dlPAG that we previously reported in *Mecp2*-het mice (Cuitavi et al., 2025). However, interestingly, fractal parameters were not significantly different for this area, which indicates a more resilient phenotype for dlPAG microglia in response to stress alterations than that of other subdivisions, similarly as previously reported for dlPAG neurons (Ho et al., 2018).

### lPAG Microglia do not Show Plasticity Changes due to Either *MeCP2* Deficiency or ELS

Unlike other PAG subdivisions, microglia at the lPAG seem more resilient to change. Although we detected a significant effect of treatment for the average number of branches per microglial cell, and an effect of both factors for endpoint voxels, these did not lead to significant pairwise comparisons (Figs. 2A3 and 3A3). Similarly, fractal analysis in this area showed no significant differences, suggesting that while morphological adaptations occur at the cellular level, these may not translate into detectable changes in global network complexity.

The findings in the lPAG, considering its integrative role in autonomic regulation and stress responses (Coulombe et al., 2016; La-Vu et al., 2022; Zhang et al., 2024), may suggest a degree of resilience in these autonomic functions against developmental deficits arising from both genetic factors (MeCP2 deficiency) and environmental influences (ELS). However, since the *Mecp2*-deficient mouse models show altered stress- and sensitivity-related characteristics even at presymptomatic stages (Abellán-Álvaro et al., 2021; Cuitavi et al., 2025) that does not seem to be the case. Thus, our lPAG data may be suggesting that another CNS area has a more prominent role governing these integrative functions, such as, for instance, the rostral ventromedial medulla, in which MeCP2 is known to modulate mechanical hypersensitivity (Imbe & Ihara, 2023). Alternatively, although unlikely, our data could be pointing that lPAG microglia is not relevant for this processing.

In any case, microglial changes at the lPAG are more subtle or require additional factors to manifest; or, as another option, lPAG microglia exhibit compensatory mechanisms not observed in other subregions. However, we could not find previous literature supporting these claims.

### Microglia from the vlPAG, Although More Resilient that in Other Subareas, Shows Altered Stress Responses in *Mecp2*-het Mice

Finally, vlPAG showed a significant interaction effect for circularity. Specifically, *Mecp2*-het mice exposed to MS demonstrated reduced circularity when compared to WT animals that also underwent MS (Fig. 5A4). This diminished circularity would be indicative of a shift towards a more ramified morphology (Vidal-Itriago et al., 2022), which suggest that microglia from *Mecp2*-hets exhibit enhanced ELS-induced ramifications than in WTs.

In addition, we also found a significant effect of the interaction in the vlPAG for lacunarity (Fig. 6A4), and an effect of treatment in terms of percentage of IBA1 (Fig. 1J). Lacunarity quantifies the spatial distribution of microglial processes, and the presence of a genotype*treatment interaction might suggest that ELS alters microglial process organisation in *Mecp2*-het mice in a manner distinct from WT controls. Although post hoc comparisons did not reach statistical significance, these findings reinforce the idea of genotype-dependent microglial plasticity within the vlPAG.

Considering vlPAG’s role, primarily involved in passive coping mechanisms (Zhang et al., 2024), our data could suggest a more nuanced form of microglial maladaptation in *Mecp2*-het mice which could manifest in fewer differences in terms of freezing behaviour or response to analgesia than in other PAG-mediated responses. However, direct evidence connecting microglial plasticity to reduced freezing behaviour is limited.

Nonetheless, in contradiction to the circularity data, no significant alterations were found in other fractal parameters or morphological analysis. This could suggest a relative resilience of microglial cells in this region to both ELS and *Mecp2* deficiency or that different mechanisms, possibly involving other glial cells or signalling pathways, govern microglial plasticity in the vlPAG. In this regard, a previous study demonstrates that repeated inescapable stress induces ΔFosB (the expression of which seems influenced by MeCP2 (Deng et al., 2010)) in the vlPAG, with the highest levels found in animals that display resilience to stress-induced behavioural deficits (Berton et al., 2007).

## Conclusion, Limitations and Ways Forward

Together, our findings highlight that *Mecp2* deficiency impairs microglial adaptability to environmental stress, with region-specific effects, within the PAG. Notably, ELS led to microglial hyper-ramification in the dmPAG and an increase in endpoints across multiple subdivisions. Although some ELS-induced changes may resemble rescue, they likely reflect a shift away from WT patterns rather than true recovery.

The failure to exhibit appropriate morphological adaptations at the PAG’s microglia, rather than overt structural loss, may account for the dysfunctional stress, defensive behaviours, anxiety, and pain processing alterations commonly observed in RTT. Accordingly, altered microglial morphology in this region may influence nociceptive signalling, potentially contributing to the pain-related phenotypes observed in RTT models (Cuitavi et al., 2025). Although we cannot determine if MeCP2 directly modulates microglial plasticity in the PAG, prior studies show that MeCP2 is expressed in microglia and can alter their function (Cronk et al., 2015; Maezawa & Jin, 2010).

Future research should explore strategies, whether pharmacological (Ishiyama et al., 2019) or through other modulatory interventions, to counteract this increased susceptibility via early-life interventions. For instance, recent studies on early-life enrichment in *Mecp2*-deficient models suggest that plastic changes might persist despite rescuing behavioural alterations (Ährlund-richter et al., 2025).

Our initial global assessment of microglial coverage, cellular density, fractal dimension, and lacunarity across the PAG revealed minimal differences, except in the vlPAG. However, region-specific analyses uncovered significant genotype*treatment interactions, highlighting the need for targeted microglial assessments to detect subtle alterations. Furthermore, we did not measure serum corticosterone, although genotype-specific differences in HPA axis activity have been previously reported in these same animals (Abellán-Álvaro et al., 2021).Additionally, our study focused on presymptomatic *Mecp2*-het females, leaving open questions about how these early differences evolve at symptomatic stages. Given that both MeCP2 loss (RTT) and overexpression (*MECP2* duplication syndrome) lead to neurodevelopmental deficits, further research on microglial alterations in *Mecp2*-mutant models, including males (Torres-Pérez et al., 2022), could provide broader insights into MeCP2’s role in neurodevelopmental disorders. In addition, future work should explore potential sex-dependent mechanisms, as MeCP2 has been implicated in female vulnerability to stress-related disorders (Cosentino et al., 2023).

## Supplementary Information

Below is the link to the electronic supplementary material.

Supplementary file1 (DOCX 140 KB)

Supplementary file2 (DOCX 3481 KB)

## Data Availability

All data are included either within main text or supplementary materials of this article. Raw additional materials, including original images, can be kindly requested to the corresponding authors.
