# Supplementary material for: Altered Microglial Plasticity in the Periaqueductal Grey of Pre-Symptomatic Mecp2-Heterozygous Mice Following Early-Life Stress
Source: Neuromolecular Med. 2025 Jun 17;27(1):46. doi: 10.1007/s12017-025-08867-9 (PMC12174278; doi:10.1007/s12017-025-08867-9)
Supplement: Supplementary file 1 — Supplementary file1 (DOCX 140 KB) [file 12017_2025_8867_MOESM1_ESM.docx]

**Supplementary materials**

**Supplementary figure 1.** **Maximum branch length per cell (one of the morphological measures taken) of the microglial cells (IBA1-ir) from the PAG.** **A)** PAG (average of the four main subdivisions). **A1)** dmPAG, **A2)** dlPAG, **A3)** lPAG, and **A4)** vlPAG. Graphs show individual values (black circles: animals subjected to standard care -SC; white squares: animals subjected to maternal separation -MS) and mean ± SEM from both wildtype (WT) and Mecp2-heterozygous (Mecp2-het) animals.

**🡨 Supplementary figure 2. Additional fractal microglial measurement from the PAG.** **A)** Cellular density, **B)** Span ratio and **C)** fractal dimension (D_β_) from the PAG. Similar data from the four major subdivisions are presented as: **A1-C1** dmPAG, **A2-C2** dlPAG, **A3-C3** lPAG, and **A4-C4** vlPAG. Graphs show individual values (black circles: animals subjected to standard care -SC; white squares: animals subjected to maternal separation -MS) and mean ± SEM from both wildtype (WT) and *Mecp2*-heterozygous (*Mecp2*-het) animals.

**Supplementary table 1. Data on the percentage of IBA1-ir**

| Animals (ref. number) | Genotype | Treatment | Branches | End-points_voxels | Average_Branch_Length | Maximum_Branch_Lenght |
| --- | --- | --- | --- | --- | --- | --- |
| M470 | WT | SC | 2,55 | 3 | 10051 | 13344 |
| M475 | WT | SC | 2,614 | 3 | 10170 | 13181 |
| M476 | WT | SC | 2,415 | 3 | 10559 | 13335 |
| M479 | WT | SC | - | - | - | - |
| M430 | WT | MS | 3,259 | 3 | 10692 | 14124 |
| M433 | WT | MS | 2,8 | 3 | 10011 | 12912 |
| M460 | WT | MS | 2,483 | 3 | 9486 | 12934 |
| M461 | WT | MS | 2,8 | 3 | 9637 | 12863 |
| M531 | WT | MS | 8,657 | 5 | 7808 | 12744 |
| M532 | WT | MS | - | - | - | - |
| M428 | HET | SC | 2,881 | 3 | 9054 | 12517 |
| M429 | HET | SC | 3,239 | 3 | 9563 | 13459 |
| M467 | HET | SC | 2,517 | 3 | 9514 | 12277 |
| M471 | HET | SC | 2,194 | 2 | 10416 | 11894 |
| M477 | HET | SC | 1,583 | 2 | 10152 | 12339 |
| M432 | HET | MS | 2,273 | 3 | 11062 | 13685 |
| M434 | HET | MS | 2,601 | 3 | 9601 | 12505 |
| M458 | HET | MS | 2,775 | 3 | 10138 | 13085 |
| M459 | HET | MS | 2,481 | 3 | 10906 | 13714 |

Table descriptors: WT: wild-type, HET: *Mecp2*-heterozygous (*Mecp2*-het), MS: maternal separation, SC: standard care.

**Supplementary table 2. Data on morphometric analysis**

| Area | Animals (ref. number) | Genotype | Treatment | Branches | End-points_voxels | Average_Branch_Length | Maximum_Branch_Lenght |
| --- | --- | --- | --- | --- | --- | --- | --- |
| PAG | M470 | WT | SC | 2,8255 | 3 | 10152,5 | 13362,25 |
|  | M475 | WT | SC | 2,6735 | 3 | 10563,375 | 13780,875 |
|  | M476 | WT | SC | 2,5075 | 3 | 11561 | 14734,25 |
|  | M479 | WT | SC | 3,2391 | 3 | 12591 | 19488 |
|  | M430 | WT | MS | 3,1405 | 3 | 10405,125 | 14085,375 |
|  | M433 | WT | MS | 3,179 | 3,25 | 10511,125 | 14189,125 |
|  | M460 | WT | MS | 3,04275 | 3 | 10686,125 | 15109,375 |
|  | M461 | WT | MS | 3,3775 | 3,25 | 9731,125 | 14267,75 |
|  | M531 | WT | MS | 5,85925 | 3,875 | 8719,375 | 12518 |
|  | M532 | WT | MS | 3,09933333 | 3 | 9487,66667 | 12799 |
|  | M428 | HET | SC | 3,001 | 2,75 | 9067,5 | 12761,75 |
|  | M429 | HET | SC | 2,56975 | 2,75 | 11366,75 | 15459,75 |
|  | M467 | HET | SC | 2,95575 | 2,75 | 10894 | 14604,875 |
|  | M471 | HET | SC | 2,528 | 2,75 | 11895,375 | 14632,25 |
|  | M477 | HET | SC | 1,964 | 2,375 | 10554,625 | 12789,25 |
|  | M432 | HET | MS | 2,7165 | 3 | 10350,625 | 13833,25 |
|  | M434 | HET | MS | 3,1515 | 3,25 | 10751,75 | 14940,25 |
|  | M458 | HET | MS | 2,977 | 3 | 10715 | 14617,75 |
|  | M459 | HET | MS | 3,284 | 3,25 | 11606,625 | 16060,375 |
| dmPAG | M470 | WT | SC | 3,216 | 3 | 9816 | 13618 |
|  | M475 | WT | SC | 3,19 | 3 | 10557 | 14219 |
|  | M476 | WT | SC | 2,642 | 3 | 12408 | 15550 |
|  | M479 | WT | SC | - | - | - | - |
|  | M430 | WT | MS | 3,02 | 3 | 10569 | 14293 |
|  | M433 | WT | MS | 4,5 | 4 | 9382 | 14665 |
|  | M460 | WT | MS | 3,485 | 3 | 12915 | 19834 |
|  | M461 | WT | MS | 3,845 | 3 | 9284 | 15342 |
|  | M531 | WT | MS | 3,088 | 3 | 9185 | 12277 |
|  | M532 | WT | MS | 3,088 | 3 | 9185 | 12277 |
|  | M428 | HET | SC | 3,182 | 3 | 10905 | 15260 |
|  | M429 | HET | SC | 2 | 2 | 14686 | 18320 |
|  | M467 | HET | SC | 2,647 | 2 | 12354 | 16061 |
|  | M471 | HET | SC | 2,423 | 3 | 13909 | 17415 |
|  | M477 | HET | SC | 1,588 | 2 | 12051 | 13635 |
|  | M432 | HET | MS | 2,611 | 3 | 10446 | 13498 |
|  | M434 | HET | MS | 4,315 | 4 | 12847 | 19062 |
|  | M458 | HET | MS | 3,36 | 3 | 11375 | 17562 |
|  | M459 | HET | MS | 4,774 | 4 | 13547 | 20666 |
| dlPAG | M470 | WT | SC | 2,781 | 3 | 10610,5 | 13527,5 |
|  | M475 | WT | SC | 2,367 | 3 | 10164,5 | 12641,5 |
|  | M476 | WT | SC | 2,15 | 3 | 10993 | 13699 |
|  | M479 | WT | SC | 3,2391 | 3 | 12591 | 19488 |
|  | M430 | WT | MS | 3,182 | 3 | 10050 | 13852 |
|  | M433 | WT | MS | 2,814 | 3 | 9490,5 | 12402,5 |
|  | M460 | WT | MS | 2,913 | 3 | 9895,5 | 12708,5 |
|  | M461 | WT | MS | 2,728 | 3 | 9669,5 | 12592 |
|  | M531 | WT | MS | 7,909 | 4,5 | 8657 | 12581 |
|  | M532 | WT | MS | 3,358 | 3 | 9581 | 13275 |
|  | M428 | HET | SC | 2,941 | 3 | 9821 | 13275 |
|  | M429 | HET | SC | 3,006 | 3 | 9596 | 13286 |
|  | M467 | HET | SC | 3,338 | 3 | 10119 | 13573,5 |
|  | M471 | HET | SC | 3,028 | 3 | 10256,5 | 12930 |
|  | M477 | HET | SC | 2,042 | 2,5 | 8782,5 | 11020 |
|  | M432 | HET | MS | 2,691 | 3 | 9862,5 | 12915 |
|  | M434 | HET | MS | 2,399 | 3 | 10527 | 12959 |
|  | M458 | HET | MS | 2,553 | 3 | 9836 | 12354 |
|  | M459 | HET | MS | 3,004 | 3 | 9891,5 | 13045,5 |
| lPAG | M470 | WT | SC | 2,755 | 3 | 10132,5 | 12959,5 |
|  | M475 | WT | SC | 2,523 | 3 | 11362 | 15082 |
|  | M476 | WT | SC | 2,823 | 3 | 12284 | 16353 |
|  | M479 | WT | SC | 2,2471 | 3 | 13454,5 | 17940 |
|  | M430 | WT | MS | 3,101 | 3 | 10309,5 | 14072,5 |
|  | M433 | WT | MS | 2,602 | 3 | 13161 | 16777 |
|  | M460 | WT | MS | 3,29 | 3 | 10448 | 14961 |
|  | M461 | WT | MS | 4,137 | 4 | 10334 | 16274 |
|  | M531 | WT | MS | 3,783 | 3 | 9227,5 | 12470 |
|  | M532 | WT | MS | 2,852 | 3 | 9697 | 12845 |
|  | M428 | HET | SC | 3 | 2 | 6490 | 9995 |
|  | M429 | HET | SC | 2,034 | 3 | 11622 | 16774 |
|  | M467 | HET | SC | 3,321 | 3 | 11589 | 16508 |
|  | M471 | HET | SC | 2,467 | 3 | 13000 | 16290 |
|  | M477 | HET | SC | 2,643 | 3 | 11233 | 14163 |
|  | M432 | HET | MS | 3,291 | 3 | 10032 | 15235 |
|  | M434 | HET | MS | 3,291 | 3 | 10032 | 15235 |
|  | M458 | HET | MS | 3,22 | 3 | 11511 | 15470 |
|  | M459 | HET | MS | 2,877 | 3 | 12082 | 16816 |
| vlPAG | M470 | WT | SC | 2,55 | 3 | 10051 | 13344 |
|  | M475 | WT | SC | 2,614 | 3 | 10170 | 13181 |
|  | M476 | WT | SC | 2,415 | 3 | 10559 | 13335 |
|  | M479 | WT | SC | - | - | - | - |
|  | M430 | WT | MS | 3,259 | 3 | 10692 | 14124 |
|  | M433 | WT | MS | 2,8 | 3 | 10011 | 12912 |
|  | M460 | WT | MS | 2,483 | 3 | 9486 | 12934 |
|  | M461 | WT | MS | 2,8 | 3 | 9637 | 12863 |
|  | M531 | WT | MS | 8,657 | 5 | 7808 | 12744 |
|  | M532 | WT | MS | - | - | - | - |
|  | M428 | HET | SC | 2,881 | 3 | 9054 | 12517 |
|  | M429 | HET | SC | 3,239 | 3 | 9563 | 13459 |
|  | M467 | HET | SC | 2,517 | 3 | 9514 | 12277 |
|  | M471 | HET | SC | 2,194 | 2 | 10416 | 11894 |
|  | M477 | HET | SC | 1,583 | 2 | 10152 | 12339 |
|  | M432 | HET | MS | 2,273 | 3 | 11062 | 13685 |
|  | M434 | HET | MS | 2,601 | 3 | 9601 | 12505 |
|  | M458 | HET | MS | 2,775 | 3 | 10138 | 13085 |
|  | M459 | HET | MS | 2,481 | 3 | 10906 | 13714 |

Table descriptors: WT: wild-type, HET: *Mecp2*-heterozygous (*Mecp2*-het), MS: maternal separation, SC: standard care.

**Supplementary table 3. Data on fractal analysis**

| Area | Animals (ref. number) | Genotype | Treatment | Density | Span_Ratio | Circularity | Fractal_Dimension_Db | Lacunarity |
| --- | --- | --- | --- | --- | --- | --- | --- | --- |
| PAG | M470 | WT | SC | 0,1346625 | 1,5201375 | 0,7917625 | 1,3212875 | 0,4273 |
|  | M475 | WT | SC | 0,0861625 | 1,4806625 | 0,7901375 | 1,282425 | 0,3623625 |
|  | M476 | WT | SC | 0,0809125 | 1,5826375 | 0,747775 | 1,2619375 | 0,3688 |
|  | M479 | WT | SC | 0,0463 | 1,428575 | 0,8004 | 1,281625 | 0,33885 |
|  | M430 | WT | MS | 0,1616875 | 1,5697375 | 0,7944875 | 1,4125 | 0,3717875 |
|  | M433 | WT | MS | 0,0869875 | 1,4828125 | 0,7598625 | 1,22825 | 0,3830125 |
|  | M460 | WT | MS | 0,0958 | 1,655025 | 0,7434625 | 1,291375 | 0,3760625 |
|  | M461 | WT | MS | 0,08511667 | 1,8405 | 0,75431667 | 1,26063333 | 0,53868333 |
|  | M531 | WT | MS | 0,1410375 | 1,5673625 | 0,7628875 | 1,221075 | 0,4216 |
|  | M532 | WT | MS | 0,14216667 | 1,44018333 | 0,78221667 | 1,31513333 | 0,41645 |
|  | M428 | HET | SC | 0,0870075 | 1,3769 | 0,8387875 | 1,4177375 | 0,4346125 |
|  | M429 | HET | SC | 0,105825 | 1,7470875 | 0,7762875 | 1,31645 | 0,3796125 |
|  | M467 | HET | SC | 0,091775 | 1,5693375 | 0,7879875 | 1,2909 | 0,47095 |
|  | M471 | HET | SC | 0,0809375 | 1,7244875 | 0,7543625 | 1,2776125 | 0,38495 |
|  | M477 | HET | SC | 0,067975 | 1,6671125 | 0,7815375 | 1,290025 | 0,396775 |
|  | M432 | HET | MS | 0,0929375 | 1,6148125 | 0,72275 | 1,27055 | 0,427975 |
|  | M434 | HET | MS | 0,0883875 | 1,558025 | 0,783325 | 1,2815125 | 0,3922625 |
|  | M458 | HET | MS | 0,0825 | 1,9503625 | 0,67225 | 1,236225 | 0,3894375 |
|  | M459 | HET | MS | 0,077475 | 1,839575 | 0,67395 | 1,27075 | 0,365775 |
| dmPAG | M470 | WT | SC | 0,0996 | 1,921 | 0,7449 | 1,2949 | 0,4543 |
|  | M475 | WT | SC | 0,0704 | 1,4787 | 0,7935 | 1,2823 | 0,3501 |
|  | M476 | WT | SC | 0,0441 | 1,7759 | 0,6978 | 1,2049 | 0,3359 |
|  | M479 | WT | SC | 0,0503 | 1,5057 | 0,7974 | 1,3165 | 0,3163 |
|  | M430 | WT | MS | 0,1459 | 1,2418 | 0,8292 | 1,3833 | 0,3596 |
|  | M433 | WT | MS | 0,0453 | 1,0988 | 0,7546 | 1,1889 | 0,4154 |
|  | M460 | WT | MS | 0,0718 | 1,6277 | 0,6765 | 1,2323 | 0,3196 |
|  | M461 | WT | MS | - | - | - | - | - |
|  | M531 | WT | MS | 0,144 | 1,0144 | 0,8025 | 1,1836 | 0,4565 |
|  | M532 | WT | MS | 0,0997 | 1,5431 | 0,6851 | 1,2732 | 0,403 |
|  | M428 | HET | SC | 0,08318 | 1,6024 | 0,8318 | 1,8256 | 0,3632 |
|  | M429 | HET | SC | 0,0434 | 1,6293 | 0,7961 | 1,258 | 0,4038 |
|  | M467 | HET | SC | 0,0689 | 1,3138 | 0,8331 | 1,2928 | 0,3809 |
|  | M471 | HET | SC | 0,0547 | 1,7776 | 0,6866 | 1,2651 | 0,3217 |
|  | M477 | HET | SC | 0,0548 | 1,6229 | 0,8089 | 1,2239 | 0,3265 |
|  | M432 | HET | MS | 0,041 | 1,7682 | 0,6999 | 1,2832 | 0,4123 |
|  | M434 | HET | MS | 0,0574 | 1,3062 | 0,8362 | 1,3434 | 0,3596 |
|  | M458 | HET | MS | 0,0657 | 2,6336 | 0,5102 | 1,2199 | 0,3325 |
|  | M459 | HET | MS | 0,0657 | 2,6336 | 0,5102 | 1,2199 | 0,3325 |
| dlPAG | M470 | WT | SC | 0,1265 | 1,4252 | 0,82445 | 1,32645 | 0,4165 |
|  | M475 | WT | SC | 0,0954 | 1,3514 | 0,82985 | 1,2447 | 0,351 |
|  | M476 | WT | SC | 0,1122 | 1,4964 | 0,7613 | 1,2488 | 0,4464 |
|  | M479 | WT | SC | - | - | - | - | - |
|  | M430 | WT | MS | 0,16425 | 2,185 | 0,67405 | 1,38405 | 0,3666 |
|  | M433 | WT | MS | 0,12315 | 2,08175 | 0,7124 | 1,23745 | 0,4092 |
|  | M460 | WT | MS | 0,13325 | 2,5188 | 0,70085 | 1,3497 | 0,35605 |
|  | M461 | WT | MS | 0,12425 | 1,304 | 0,83465 | 1,27515 | 0,4114 |
|  | M531 | WT | MS | 0,1632 | 1,7526 | 0,736 | 1,1847 | 0,36915 |
|  | M532 | WT | MS | 0,1552 | 1,28855 | 0,8708 | 1,34025 | 0,4161 |
|  | M428 | HET | SC | 0,1095 | 1,3502 | 0,83385 | 1,2994 | 0,42255 |
|  | M429 | HET | SC | 0,14875 | 1,9053 | 0,74735 | 1,35235 | 0,35365 |
|  | M467 | HET | SC | 0,09705 | 1,32815 | 0,8183 | 1,30085 | 0,62325 |
|  | M471 | HET | SC | 0,1279 | 1,2135 | 0,86185 | 1,3679 | 0,38035 |
|  | M477 | HET | SC | 0,06825 | 1,4634 | 0,84625 | 1,31725 | 0,4482 |
|  | M432 | HET | MS | 0,1098 | 1,16275 | 0,7918 | 1,25115 | 0,4243 |
|  | M434 | HET | MS | 0,113 | 1,88955 | 0,7719 | 1,28115 | 0,4589 |
|  | M458 | HET | MS | 0,1201 | 1,4247 | 0,76375 | 1,25975 | 0,42395 |
|  | M459 | HET | MS | 0,1275 | 1,5788 | 0,7533 | 1,3392 | 0,4021 |
| lPAG | M470 | WT | SC | 0,12785 | 1,15005 | 0,8725 | 1,3083 | 0,4574 |
|  | M475 | WT | SC | 0,06215 | 1,95055 | 0,7233 | 1,2814 | 0,33265 |
|  | M476 | WT | SC | 0,04675 | 1,71955 | 0,7246 | 1,27795 | 0,3314 |
|  | M479 | WT | SC | 0,0423 | 1,35145 | 0,8034 | 1,24675 | 0,3614 |
|  | M430 | WT | MS | 0,1528 | 1,62465 | 0,7871 | 1,38515 | 0,40485 |
|  | M433 | WT | MS | 0,0586 | 1,2569 | 0,79015 | 1,18645 | 0,37485 |
|  | M460 | WT | MS | 0,04675 | 1,3961 | 0,7508 | 1,2499 | 0,3677 |
|  | M461 | WT | MS | 0,057 | 2,8855 | 0,5538 | 1,26345 | 0,34115 |
|  | M531 | WT | MS | 0,13985 | 1,69965 | 0,76995 | 1,2977 | 0,38545 |
|  | M532 | WT | MS | 0,1716 | 1,4889 | 0,79075 | 1,33195 | 0,43025 |
|  | M428 | HET | SC | 0,04485 | 1,3794 | 0,827 | 1,22625 | 0,4584 |
|  | M429 | HET | SC | 0,07755 | 2,05575 | 0,7176 | 1,30055 | 0,2523 |
|  | M467 | HET | SC | 0,05765 | 2,3086 | 0,68545 | 1,24395 | 0,42195 |
|  | M471 | HET | SC | 0,04825 | 2,00035 | 0,7052 | 1,26355 | 0,38395 |
|  | M477 | HET | SC | 0,06845 | 1,36935 | 0,7823 | 1,31835 | 0,3343 |
|  | M432 | HET | MS | 0,09455 | 1,4365 | 0,7509 | 1,31335 | 0,5162 |
|  | M434 | HET | MS | 0,05905 | 1,51015 | 0,7924 | 1,2499 | 0,37245 |
|  | M458 | HET | MS | 0,0464 | 1,36345 | 0,73585 | 1,23725 | 0,436 |
|  | M459 | HET | MS | 0,0464 | 1,8899 | 0,699 | 1,3028 | 0,3461 |
| vlPAG | M470 | WT | SC | 0,1847 | 1,5843 | 0,7252 | 1,3555 | 0,381 |
|  | M475 | WT | SC | 0,1167 | 1,142 | 0,8139 | 1,3213 | 0,4157 |
|  | M476 | WT | SC | 0,1206 | 1,3387 | 0,8074 | 1,3161 | 0,3615 |
|  | M479 | WT | SC | - | - | - | - | - |
|  | M430 | WT | MS | 0,1838 | 1,2275 | 0,8876 | 1,4975 | 0,3561 |
|  | M433 | WT | MS | 0,1209 | 1,4938 | 0,7823 | 1,3002 | 0,3326 |
|  | M460 | WT | MS | 0,1314 | 1,0775 | 0,8457 | 1,3336 | 0,4609 |
|  | M461 | WT | MS | 0,0741 | 1,332 | 0,8745 | 1,2433 | 0,8635 |
|  | M531 | WT | MS | 0,1171 | 1,8028 | 0,7431 | 1,2183 | 0,4753 |
|  | M532 | WT | MS | - | - | - | - | - |
|  | M428 | HET | SC | 0,1105 | 1,1756 | 0,8625 | 1,3197 | 0,4943 |
|  | M429 | HET | SC | 0,1536 | 1,398 | 0,8441 | 1,3549 | 0,5087 |
|  | M467 | HET | SC | 0,1435 | 1,3268 | 0,8151 | 1,326 | 0,4577 |
|  | M471 | HET | SC | 0,0929 | 1,9065 | 0,7638 | 1,2139 | 0,4538 |
|  | M477 | HET | SC | 0,0804 | 2,2128 | 0,6887 | 1,3006 | 0,4781 |
|  | M432 | HET | MS | 0,1264 | 2,0918 | 0,6484 | 1,2345 | 0,3591 |
|  | M434 | HET | MS | 0,1241 | 1,5262 | 0,7328 | 1,2516 | 0,3781 |
|  | M458 | HET | MS | 0,0978 | 2,3797 | 0,6792 | 1,228 | 0,3653 |
|  | M459 | HET | MS | 0,0703 | 1,256 | 0,7333 | 1,2211 | 0,3824 |

Table descriptors: WT: wild-type, HET: *Mecp2*-heterozygous (*Mecp2*-het), MS: maternal separation, SC: standard care.

**# Supplemental Material: Statistical Analysis in R**

# 1. Load Required Packages

library(tidyverse) # Data manipulation

library(ARTool) # Aligned Rank Transformation ANOVA

library(car) # Levene’s test

library(rcompanion) # Post-hoc analysis

library(readxl) # Reading Excel files

# 2. Data Preprocessing

file_path <- "your_data_file.xlsx" # Update with actual file path

sheet_name <- "your_sheet_name" # Update with actual sheet name

df <- read_excel(file_path, sheet = sheet_name)

# Convert "-" to NA and ensure numerical variables are correctly formatted

numeric_vars <- c("Variable_1", "Variable_2", "Variable_3") # Replace with actual variable names

df[df == "-"] <- NA

df[numeric_vars] <- lapply(df[numeric_vars], as.numeric)

df <- na.omit(df)

# Convert categorical variables to factors

df$Genotype <- as.factor(df$Genotype)

df$Treatment <- as.factor(df$Treatment)

# 3. Normality and Homoscedasticity Tests

shapiro_results <- sapply(numeric_vars, function(var) shapiro.test(df[[var]])$p.value)

levene_results <- sapply(numeric_vars, function(var) {

leveneTest(df[[var]] ~ Genotype * Treatment, data = df)$"Pr(>F)"[1]

})

# Identify variable groups based on test results

anova_vars <- names(which(shapiro_results > 0.05 & levene_results > 0.05)) # Normal & homoscedastic

art_vars <- names(which(shapiro_results <= 0.05)) # Non-normal data

# 4. Statistical Analysis

## Two-way ANOVA for Normally Distributed Variables

anova_models <- lapply(anova_vars, function(var) {

anova_formula <- as.formula(paste(var, "~ Genotype * Treatment"))

aov(anova_formula, data = df)

})

names(anova_models) <- anova_vars

# Get ANOVA results

anova_results <- lapply(anova_models, summary)

anova_results

## Post-hoc Tests for Two-way ANOVA (Bonferroni Correction)

anova_posthoc <- lapply(anova_vars, function(var) {

pairwise.t.test(df[[var]], interaction(df$Genotype, df$Treatment), p.adjust.method = "bonferroni")

})

names(anova_posthoc) <- anova_vars

## ART ANOVA for Non-Normal Variables

art_models <- lapply(art_vars, function(var) {

art_formula <- as.formula(paste(var, "~ Genotype * Treatment"))

art(art_formula, data = df)

})

names(art_models) <- art_vars

# Get ART ANOVA results

art_anova_results <- lapply(art_models, anova)

art_anova_results

## Post-hoc Tests for ART ANOVA (Bonferroni Correction)

art_posthoc <- lapply(art_vars, function(var) {

pairwise.t.test(df[[var]], interaction(df$Genotype, df$Treatment), p.adjust.method = "bonferroni")

})

names(art_posthoc) <- art_vars

# 5. Summary of Statistical Tests Applied

cat("\nVariables analyzed with Two-way ANOVA:", anova_vars)

cat("\nVariables analyzed with ART ANOVA:", art_vars)

# For all tests, Bonferroni correction was applied to post-hoc comparisons.
