# Supplementary material for: Altered Microglial Plasticity in the Periaqueductal Grey of Pre-Symptomatic Mecp2-Heterozygous Mice Following Early-Life Stress
Source: Neuromolecular Med. 2025 Jun 17;27(1):46. doi: 10.1007/s12017-025-08867-9 (PMC12174278; doi:10.1007/s12017-025-08867-9)
Supplement: Supplementary file 2 — Supplementary file2 (DOCX 3481 KB) [file 12017_2025_8867_MOESM2_ESM.docx]

**Supplementary materials**


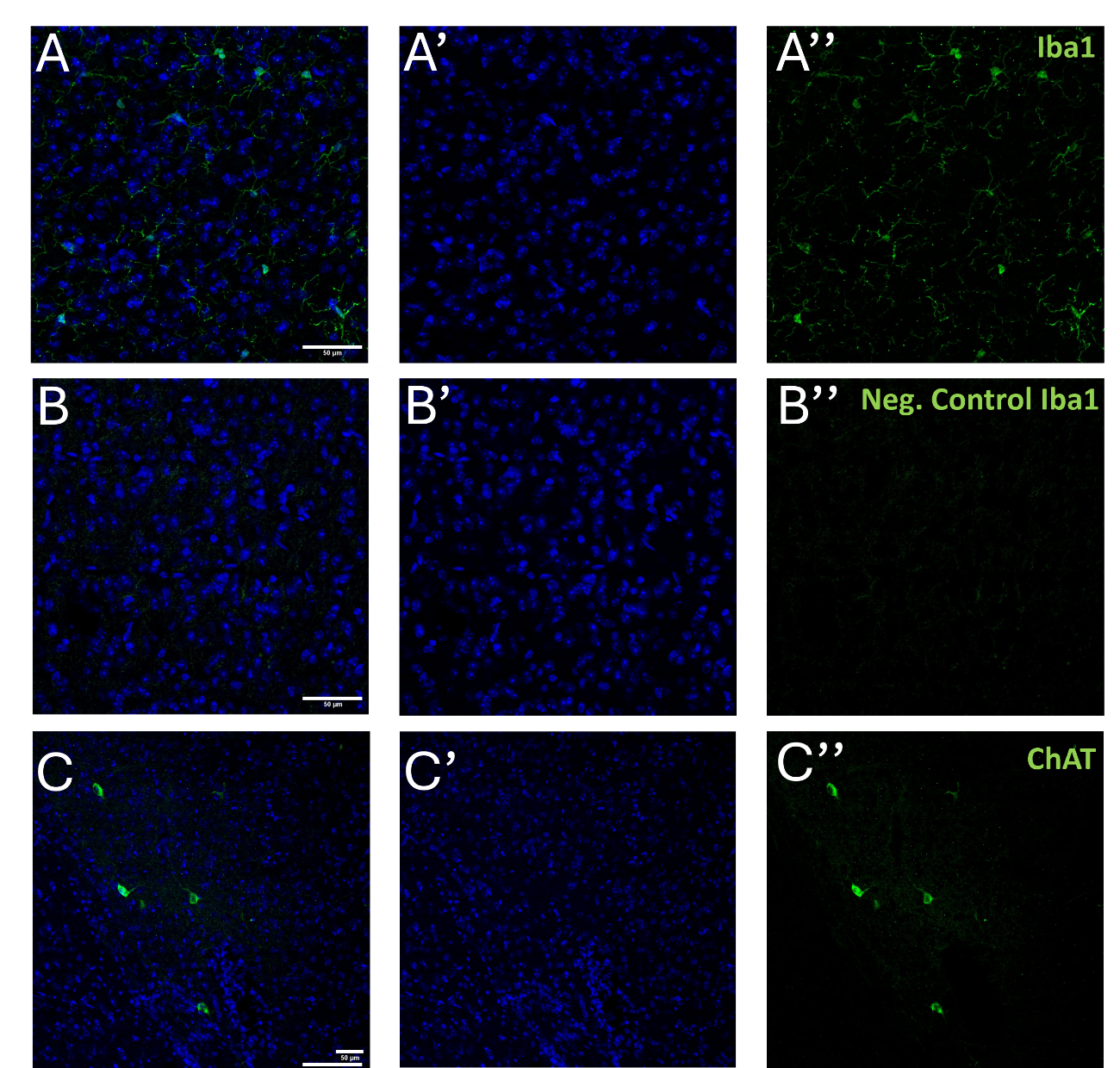


**Supplementary Figure 1. Negative and positive controls for IBA1 immunofluorescence in the PAG. A)** dlPAG from one of the animals used in the study (ref: M467) processed with the primary anti-IBA1 antibody (green) and DAPI (blue). **B)** Negative control: dlPAG from the same animal processed without the primary anti-IBA1 antibody, showing only DAPI nuclear staining (blue). No non-specific signal is detected in the green channel, confirming staining specificity under our experimental conditions. **C)** Positive control: secondary antibody control using a different goat-derived primary (anti-ChaT, 1:200; doi: 10.3389/fnana.2016.00125), confirming absence of cross-reactivity in the IBA1 detection channel (green); image shows cholinergic interneurons in the striatum. In all cases, scale bars represent 50 µm. The anti-IBA1 antibody used (Abcam, ab5076) has also been validated by the manufacturer via western blot (single 16 kDa band) and cited in over 1300 peer-reviewed articles.

**Supplementary figure 2.** **Maximum branch length per cell (one of the morphological measures taken) of the microglial cells (IBA1-ir) from the PAG.** **A)** PAG (average of the four main subdivisions). **A1)** dmPAG, **A2)** dlPAG, **A3)** lPAG, and **A4)** vlPAG. Graphs show individual values (black circles: animals subjected to standard care -SC; white squares: animals subjected to maternal separation -MS) and mean ± SEM from both wildtype (WT) and Mecp2-heterozygous (Mecp2-het) animals.


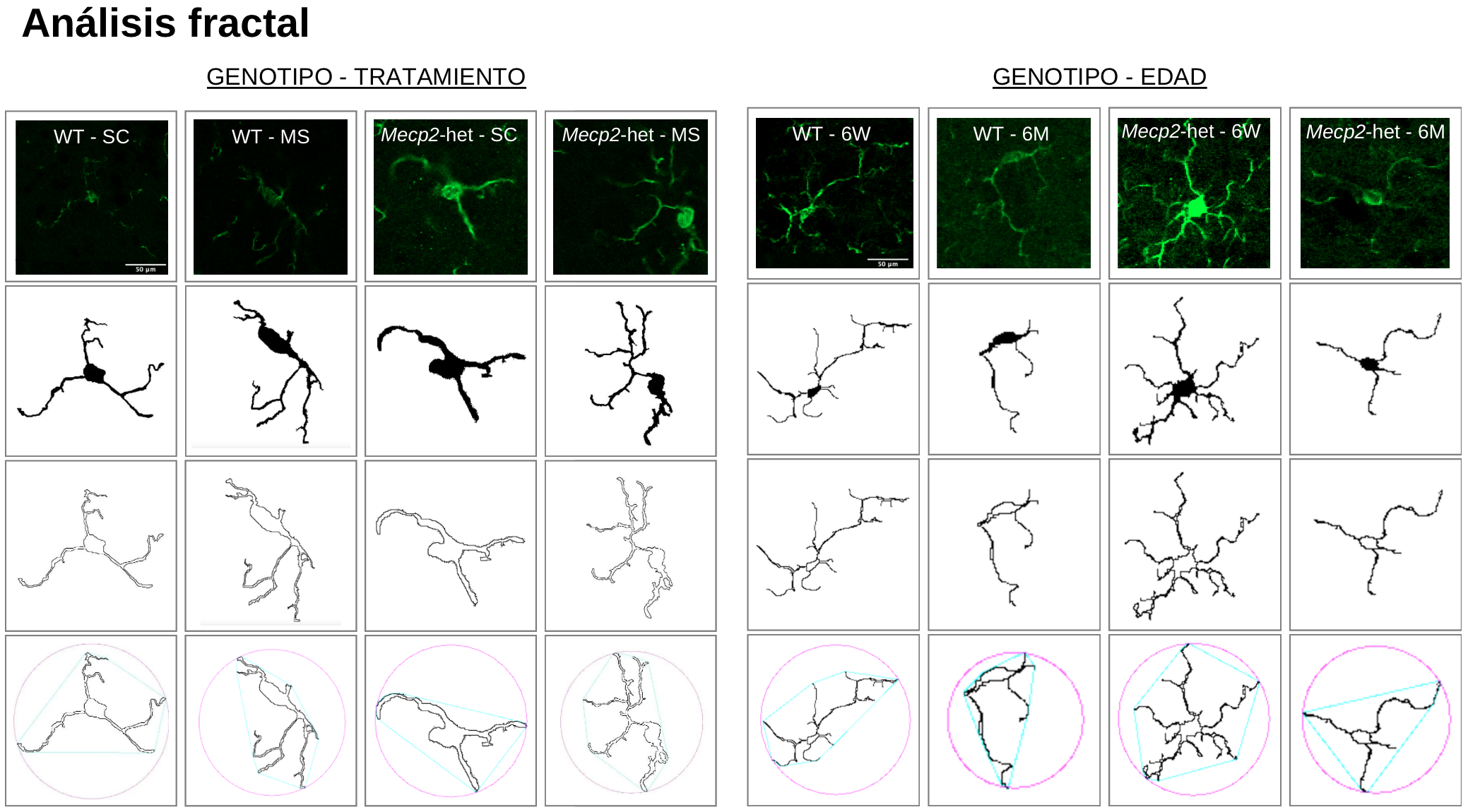


**Supplementary figure 3. Representative microglial images used for fractal analysis.** Example of cropped photomicrographs of microglia (IBA1-ir cells) in the PAG (first row) with corresponding binary (second row), resulting outline images (third row) and the outlined associated convex hull (blue) and enclosing circle (pink) for the outline shapes (fourth row). Each column represents a different experimental group: WT animals subjected to standard care (WT-SC, first column, at the left side of the figure) or maternal separation (WT-MS, second column), and *Mecp2*-heterozygous animals under standard care (Mecp2-het SC, third column) or maternal separation (Mecp2-het MS, last column, at the right side). All images at same magnification; scale bar represents 50 µm.

**🡨 Supplementary figure 4. Additional fractal microglial measurement from the PAG.** **A)** Cellular density, **B)** Span ratio and **C)** fractal dimension (D_β_) from the PAG. Similar data from the four major subdivisions are presented as: **A1-C1** dmPAG, **A2-C2** dlPAG, **A3-C3** lPAG, and **A4-C4** vlPAG. Graphs show individual values (black circles: animals subjected to standard care -SC; white squares: animals subjected to maternal separation -MS) and mean ± SEM from both wildtype (WT) and *Mecp2*-heterozygous (*Mecp2*-het) animals.

**Supplementary table 1. Data on the percentage of IBA1-ir**

| Animals (ref. number) | Genotype | Treatment | Branches | End-points_voxels | Average_Branch_Length | Maximum_Branch_Lenght |
| --- | --- | --- | --- | --- | --- | --- |
| M470 | WT | SC | 2,55 | 3 | 10051 | 13344 |
| M475 | WT | SC | 2,614 | 3 | 10170 | 13181 |
| M476 | WT | SC | 2,415 | 3 | 10559 | 13335 |
| M479 | WT | SC | - | - | - | - |
| M430 | WT | MS | 3,259 | 3 | 10692 | 14124 |
| M433 | WT | MS | 2,8 | 3 | 10011 | 12912 |
| M460 | WT | MS | 2,483 | 3 | 9486 | 12934 |
| M461 | WT | MS | 2,8 | 3 | 9637 | 12863 |
| M531 | WT | MS | 8,657 | 5 | 7808 | 12744 |
| M532 | WT | MS | - | - | - | - |
| M428 | HET | SC | 2,881 | 3 | 9054 | 12517 |
| M429 | HET | SC | 3,239 | 3 | 9563 | 13459 |
| M467 | HET | SC | 2,517 | 3 | 9514 | 12277 |
| M471 | HET | SC | 2,194 | 2 | 10416 | 11894 |
| M477 | HET | SC | 1,583 | 2 | 10152 | 12339 |
| M432 | HET | MS | 2,273 | 3 | 11062 | 13685 |
| M434 | HET | MS | 2,601 | 3 | 9601 | 12505 |
| M458 | HET | MS | 2,775 | 3 | 10138 | 13085 |
| M459 | HET | MS | 2,481 | 3 | 10906 | 13714 |

Table descriptors: WT: wild-type, HET: *Mecp2*-heterozygous (*Mecp2*-het), MS: maternal separation, SC: standard care.

**Supplementary table 2. Data on morphometric analysis**

| Area | Animals (ref. number) | Genotype | Treatment | Branches | End-points_voxels | Average_Branch_Length | Maximum_Branch_Lenght |
| --- | --- | --- | --- | --- | --- | --- | --- |
| PAG | M470 | WT | SC | 2,8255 | 3 | 10152,5 | 13362,25 |
|  | M475 | WT | SC | 2,6735 | 3 | 10563,375 | 13780,875 |
|  | M476 | WT | SC | 2,5075 | 3 | 11561 | 14734,25 |
|  | M479 | WT | SC | 3,2391 | 3 | 12591 | 19488 |
|  | M430 | WT | MS | 3,1405 | 3 | 10405,125 | 14085,375 |
|  | M433 | WT | MS | 3,179 | 3,25 | 10511,125 | 14189,125 |
|  | M460 | WT | MS | 3,04275 | 3 | 10686,125 | 15109,375 |
|  | M461 | WT | MS | 3,3775 | 3,25 | 9731,125 | 14267,75 |
|  | M531 | WT | MS | 5,85925 | 3,875 | 8719,375 | 12518 |
|  | M532 | WT | MS | 3,09933333 | 3 | 9487,66667 | 12799 |
|  | M428 | HET | SC | 3,001 | 2,75 | 9067,5 | 12761,75 |
|  | M429 | HET | SC | 2,56975 | 2,75 | 11366,75 | 15459,75 |
|  | M467 | HET | SC | 2,95575 | 2,75 | 10894 | 14604,875 |
|  | M471 | HET | SC | 2,528 | 2,75 | 11895,375 | 14632,25 |
|  | M477 | HET | SC | 1,964 | 2,375 | 10554,625 | 12789,25 |
|  | M432 | HET | MS | 2,7165 | 3 | 10350,625 | 13833,25 |
|  | M434 | HET | MS | 3,1515 | 3,25 | 10751,75 | 14940,25 |
|  | M458 | HET | MS | 2,977 | 3 | 10715 | 14617,75 |
|  | M459 | HET | MS | 3,284 | 3,25 | 11606,625 | 16060,375 |
| dmPAG | M470 | WT | SC | 3,216 | 3 | 9816 | 13618 |
|  | M475 | WT | SC | 3,19 | 3 | 10557 | 14219 |
|  | M476 | WT | SC | 2,642 | 3 | 12408 | 15550 |
|  | M479 | WT | SC | - | - | - | - |
|  | M430 | WT | MS | 3,02 | 3 | 10569 | 14293 |
|  | M433 | WT | MS | 4,5 | 4 | 9382 | 14665 |
|  | M460 | WT | MS | 3,485 | 3 | 12915 | 19834 |
|  | M461 | WT | MS | 3,845 | 3 | 9284 | 15342 |
|  | M531 | WT | MS | 3,088 | 3 | 9185 | 12277 |
|  | M532 | WT | MS | 3,088 | 3 | 9185 | 12277 |
|  | M428 | HET | SC | 3,182 | 3 | 10905 | 15260 |
|  | M429 | HET | SC | 2 | 2 | 14686 | 18320 |
|  | M467 | HET | SC | 2,647 | 2 | 12354 | 16061 |
|  | M471 | HET | SC | 2,423 | 3 | 13909 | 17415 |
|  | M477 | HET | SC | 1,588 | 2 | 12051 | 13635 |
|  | M432 | HET | MS | 2,611 | 3 | 10446 | 13498 |
|  | M434 | HET | MS | 4,315 | 4 | 12847 | 19062 |
|  | M458 | HET | MS | 3,36 | 3 | 11375 | 17562 |
|  | M459 | HET | MS | 4,774 | 4 | 13547 | 20666 |
| dlPAG | M470 | WT | SC | 2,781 | 3 | 10610,5 | 13527,5 |
|  | M475 | WT | SC | 2,367 | 3 | 10164,5 | 12641,5 |
|  | M476 | WT | SC | 2,15 | 3 | 10993 | 13699 |
|  | M479 | WT | SC | 3,2391 | 3 | 12591 | 19488 |
|  | M430 | WT | MS | 3,182 | 3 | 10050 | 13852 |
|  | M433 | WT | MS | 2,814 | 3 | 9490,5 | 12402,5 |
|  | M460 | WT | MS | 2,913 | 3 | 9895,5 | 12708,5 |
|  | M461 | WT | MS | 2,728 | 3 | 9669,5 | 12592 |
|  | M531 | WT | MS | 7,909 | 4,5 | 8657 | 12581 |
|  | M532 | WT | MS | 3,358 | 3 | 9581 | 13275 |
|  | M428 | HET | SC | 2,941 | 3 | 9821 | 13275 |
|  | M429 | HET | SC | 3,006 | 3 | 9596 | 13286 |
|  | M467 | HET | SC | 3,338 | 3 | 10119 | 13573,5 |
|  | M471 | HET | SC | 3,028 | 3 | 10256,5 | 12930 |
|  | M477 | HET | SC | 2,042 | 2,5 | 8782,5 | 11020 |
|  | M432 | HET | MS | 2,691 | 3 | 9862,5 | 12915 |
|  | M434 | HET | MS | 2,399 | 3 | 10527 | 12959 |
|  | M458 | HET | MS | 2,553 | 3 | 9836 | 12354 |
|  | M459 | HET | MS | 3,004 | 3 | 9891,5 | 13045,5 |
| lPAG | M470 | WT | SC | 2,755 | 3 | 10132,5 | 12959,5 |
|  | M475 | WT | SC | 2,523 | 3 | 11362 | 15082 |
|  | M476 | WT | SC | 2,823 | 3 | 12284 | 16353 |
|  | M479 | WT | SC | 2,2471 | 3 | 13454,5 | 17940 |
|  | M430 | WT | MS | 3,101 | 3 | 10309,5 | 14072,5 |
|  | M433 | WT | MS | 2,602 | 3 | 13161 | 16777 |
|  | M460 | WT | MS | 3,29 | 3 | 10448 | 14961 |
|  | M461 | WT | MS | 4,137 | 4 | 10334 | 16274 |
|  | M531 | WT | MS | 3,783 | 3 | 9227,5 | 12470 |
|  | M532 | WT | MS | 2,852 | 3 | 9697 | 12845 |
|  | M428 | HET | SC | 3 | 2 | 6490 | 9995 |
|  | M429 | HET | SC | 2,034 | 3 | 11622 | 16774 |
|  | M467 | HET | SC | 3,321 | 3 | 11589 | 16508 |
|  | M471 | HET | SC | 2,467 | 3 | 13000 | 16290 |
|  | M477 | HET | SC | 2,643 | 3 | 11233 | 14163 |
|  | M432 | HET | MS | 3,291 | 3 | 10032 | 15235 |
|  | M434 | HET | MS | 3,291 | 3 | 10032 | 15235 |
|  | M458 | HET | MS | 3,22 | 3 | 11511 | 15470 |
|  | M459 | HET | MS | 2,877 | 3 | 12082 | 16816 |
| vlPAG | M470 | WT | SC | 2,55 | 3 | 10051 | 13344 |
|  | M475 | WT | SC | 2,614 | 3 | 10170 | 13181 |
|  | M476 | WT | SC | 2,415 | 3 | 10559 | 13335 |
|  | M479 | WT | SC | - | - | - | - |
|  | M430 | WT | MS | 3,259 | 3 | 10692 | 14124 |
|  | M433 | WT | MS | 2,8 | 3 | 10011 | 12912 |
|  | M460 | WT | MS | 2,483 | 3 | 9486 | 12934 |
|  | M461 | WT | MS | 2,8 | 3 | 9637 | 12863 |
|  | M531 | WT | MS | 8,657 | 5 | 7808 | 12744 |
|  | M532 | WT | MS | - | - | - | - |
|  | M428 | HET | SC | 2,881 | 3 | 9054 | 12517 |
|  | M429 | HET | SC | 3,239 | 3 | 9563 | 13459 |
|  | M467 | HET | SC | 2,517 | 3 | 9514 | 12277 |
|  | M471 | HET | SC | 2,194 | 2 | 10416 | 11894 |
|  | M477 | HET | SC | 1,583 | 2 | 10152 | 12339 |
|  | M432 | HET | MS | 2,273 | 3 | 11062 | 13685 |
|  | M434 | HET | MS | 2,601 | 3 | 9601 | 12505 |
|  | M458 | HET | MS | 2,775 | 3 | 10138 | 13085 |
|  | M459 | HET | MS | 2,481 | 3 | 10906 | 13714 |

Table descriptors: WT: wild-type, HET: *Mecp2*-heterozygous (*Mecp2*-het), MS: maternal separation, SC: standard care.

**Supplementary table 3. Data on fractal analysis**

| Area | Animals (ref. number) | Genotype | Treatment | Density | Span_Ratio | Circularity | Fractal_Dimension_Db | Lacunarity |
| --- | --- | --- | --- | --- | --- | --- | --- | --- |
| PAG | M470 | WT | SC | 0,1346625 | 1,5201375 | 0,7917625 | 1,3212875 | 0,4273 |
|  | M475 | WT | SC | 0,0861625 | 1,4806625 | 0,7901375 | 1,282425 | 0,3623625 |
|  | M476 | WT | SC | 0,0809125 | 1,5826375 | 0,747775 | 1,2619375 | 0,3688 |
|  | M479 | WT | SC | 0,0463 | 1,428575 | 0,8004 | 1,281625 | 0,33885 |
|  | M430 | WT | MS | 0,1616875 | 1,5697375 | 0,7944875 | 1,4125 | 0,3717875 |
|  | M433 | WT | MS | 0,0869875 | 1,4828125 | 0,7598625 | 1,22825 | 0,3830125 |
|  | M460 | WT | MS | 0,0958 | 1,655025 | 0,7434625 | 1,291375 | 0,3760625 |
|  | M461 | WT | MS | 0,08511667 | 1,8405 | 0,75431667 | 1,26063333 | 0,53868333 |
|  | M531 | WT | MS | 0,1410375 | 1,5673625 | 0,7628875 | 1,221075 | 0,4216 |
|  | M532 | WT | MS | 0,14216667 | 1,44018333 | 0,78221667 | 1,31513333 | 0,41645 |
|  | M428 | HET | SC | 0,0870075 | 1,3769 | 0,8387875 | 1,4177375 | 0,4346125 |
|  | M429 | HET | SC | 0,105825 | 1,7470875 | 0,7762875 | 1,31645 | 0,3796125 |
|  | M467 | HET | SC | 0,091775 | 1,5693375 | 0,7879875 | 1,2909 | 0,47095 |
|  | M471 | HET | SC | 0,0809375 | 1,7244875 | 0,7543625 | 1,2776125 | 0,38495 |
|  | M477 | HET | SC | 0,067975 | 1,6671125 | 0,7815375 | 1,290025 | 0,396775 |
|  | M432 | HET | MS | 0,0929375 | 1,6148125 | 0,72275 | 1,27055 | 0,427975 |
|  | M434 | HET | MS | 0,0883875 | 1,558025 | 0,783325 | 1,2815125 | 0,3922625 |
|  | M458 | HET | MS | 0,0825 | 1,9503625 | 0,67225 | 1,236225 | 0,3894375 |
|  | M459 | HET | MS | 0,077475 | 1,839575 | 0,67395 | 1,27075 | 0,365775 |
| dmPAG | M470 | WT | SC | 0,0996 | 1,921 | 0,7449 | 1,2949 | 0,4543 |
|  | M475 | WT | SC | 0,0704 | 1,4787 | 0,7935 | 1,2823 | 0,3501 |
|  | M476 | WT | SC | 0,0441 | 1,7759 | 0,6978 | 1,2049 | 0,3359 |
|  | M479 | WT | SC | 0,0503 | 1,5057 | 0,7974 | 1,3165 | 0,3163 |
|  | M430 | WT | MS | 0,1459 | 1,2418 | 0,8292 | 1,3833 | 0,3596 |
|  | M433 | WT | MS | 0,0453 | 1,0988 | 0,7546 | 1,1889 | 0,4154 |
|  | M460 | WT | MS | 0,0718 | 1,6277 | 0,6765 | 1,2323 | 0,3196 |
|  | M461 | WT | MS | - | - | - | - | - |
|  | M531 | WT | MS | 0,144 | 1,0144 | 0,8025 | 1,1836 | 0,4565 |
|  | M532 | WT | MS | 0,0997 | 1,5431 | 0,6851 | 1,2732 | 0,403 |
|  | M428 | HET | SC | 0,08318 | 1,6024 | 0,8318 | 1,8256 | 0,3632 |
|  | M429 | HET | SC | 0,0434 | 1,6293 | 0,7961 | 1,258 | 0,4038 |
|  | M467 | HET | SC | 0,0689 | 1,3138 | 0,8331 | 1,2928 | 0,3809 |
|  | M471 | HET | SC | 0,0547 | 1,7776 | 0,6866 | 1,2651 | 0,3217 |
|  | M477 | HET | SC | 0,0548 | 1,6229 | 0,8089 | 1,2239 | 0,3265 |
|  | M432 | HET | MS | 0,041 | 1,7682 | 0,6999 | 1,2832 | 0,4123 |
|  | M434 | HET | MS | 0,0574 | 1,3062 | 0,8362 | 1,3434 | 0,3596 |
|  | M458 | HET | MS | 0,0657 | 2,6336 | 0,5102 | 1,2199 | 0,3325 |
|  | M459 | HET | MS | 0,0657 | 2,6336 | 0,5102 | 1,2199 | 0,3325 |
| dlPAG | M470 | WT | SC | 0,1265 | 1,4252 | 0,82445 | 1,32645 | 0,4165 |
|  | M475 | WT | SC | 0,0954 | 1,3514 | 0,82985 | 1,2447 | 0,351 |
|  | M476 | WT | SC | 0,1122 | 1,4964 | 0,7613 | 1,2488 | 0,4464 |
|  | M479 | WT | SC | - | - | - | - | - |
|  | M430 | WT | MS | 0,16425 | 2,185 | 0,67405 | 1,38405 | 0,3666 |
|  | M433 | WT | MS | 0,12315 | 2,08175 | 0,7124 | 1,23745 | 0,4092 |
|  | M460 | WT | MS | 0,13325 | 2,5188 | 0,70085 | 1,3497 | 0,35605 |
|  | M461 | WT | MS | 0,12425 | 1,304 | 0,83465 | 1,27515 | 0,4114 |
|  | M531 | WT | MS | 0,1632 | 1,7526 | 0,736 | 1,1847 | 0,36915 |
|  | M532 | WT | MS | 0,1552 | 1,28855 | 0,8708 | 1,34025 | 0,4161 |
|  | M428 | HET | SC | 0,1095 | 1,3502 | 0,83385 | 1,2994 | 0,42255 |
|  | M429 | HET | SC | 0,14875 | 1,9053 | 0,74735 | 1,35235 | 0,35365 |
|  | M467 | HET | SC | 0,09705 | 1,32815 | 0,8183 | 1,30085 | 0,62325 |
|  | M471 | HET | SC | 0,1279 | 1,2135 | 0,86185 | 1,3679 | 0,38035 |
|  | M477 | HET | SC | 0,06825 | 1,4634 | 0,84625 | 1,31725 | 0,4482 |
|  | M432 | HET | MS | 0,1098 | 1,16275 | 0,7918 | 1,25115 | 0,4243 |
|  | M434 | HET | MS | 0,113 | 1,88955 | 0,7719 | 1,28115 | 0,4589 |
|  | M458 | HET | MS | 0,1201 | 1,4247 | 0,76375 | 1,25975 | 0,42395 |
|  | M459 | HET | MS | 0,1275 | 1,5788 | 0,7533 | 1,3392 | 0,4021 |
| lPAG | M470 | WT | SC | 0,12785 | 1,15005 | 0,8725 | 1,3083 | 0,4574 |
|  | M475 | WT | SC | 0,06215 | 1,95055 | 0,7233 | 1,2814 | 0,33265 |
|  | M476 | WT | SC | 0,04675 | 1,71955 | 0,7246 | 1,27795 | 0,3314 |
|  | M479 | WT | SC | 0,0423 | 1,35145 | 0,8034 | 1,24675 | 0,3614 |
|  | M430 | WT | MS | 0,1528 | 1,62465 | 0,7871 | 1,38515 | 0,40485 |
|  | M433 | WT | MS | 0,0586 | 1,2569 | 0,79015 | 1,18645 | 0,37485 |
|  | M460 | WT | MS | 0,04675 | 1,3961 | 0,7508 | 1,2499 | 0,3677 |
|  | M461 | WT | MS | 0,057 | 2,8855 | 0,5538 | 1,26345 | 0,34115 |
|  | M531 | WT | MS | 0,13985 | 1,69965 | 0,76995 | 1,2977 | 0,38545 |
|  | M532 | WT | MS | 0,1716 | 1,4889 | 0,79075 | 1,33195 | 0,43025 |
|  | M428 | HET | SC | 0,04485 | 1,3794 | 0,827 | 1,22625 | 0,4584 |
|  | M429 | HET | SC | 0,07755 | 2,05575 | 0,7176 | 1,30055 | 0,2523 |
|  | M467 | HET | SC | 0,05765 | 2,3086 | 0,68545 | 1,24395 | 0,42195 |
|  | M471 | HET | SC | 0,04825 | 2,00035 | 0,7052 | 1,26355 | 0,38395 |
|  | M477 | HET | SC | 0,06845 | 1,36935 | 0,7823 | 1,31835 | 0,3343 |
|  | M432 | HET | MS | 0,09455 | 1,4365 | 0,7509 | 1,31335 | 0,5162 |
|  | M434 | HET | MS | 0,05905 | 1,51015 | 0,7924 | 1,2499 | 0,37245 |
|  | M458 | HET | MS | 0,0464 | 1,36345 | 0,73585 | 1,23725 | 0,436 |
|  | M459 | HET | MS | 0,0464 | 1,8899 | 0,699 | 1,3028 | 0,3461 |
| vlPAG | M470 | WT | SC | 0,1847 | 1,5843 | 0,7252 | 1,3555 | 0,381 |
|  | M475 | WT | SC | 0,1167 | 1,142 | 0,8139 | 1,3213 | 0,4157 |
|  | M476 | WT | SC | 0,1206 | 1,3387 | 0,8074 | 1,3161 | 0,3615 |
|  | M479 | WT | SC | - | - | - | - | - |
|  | M430 | WT | MS | 0,1838 | 1,2275 | 0,8876 | 1,4975 | 0,3561 |
|  | M433 | WT | MS | 0,1209 | 1,4938 | 0,7823 | 1,3002 | 0,3326 |
|  | M460 | WT | MS | 0,1314 | 1,0775 | 0,8457 | 1,3336 | 0,4609 |
|  | M461 | WT | MS | 0,0741 | 1,332 | 0,8745 | 1,2433 | 0,8635 |
|  | M531 | WT | MS | 0,1171 | 1,8028 | 0,7431 | 1,2183 | 0,4753 |
|  | M532 | WT | MS | - | - | - | - | - |
|  | M428 | HET | SC | 0,1105 | 1,1756 | 0,8625 | 1,3197 | 0,4943 |
|  | M429 | HET | SC | 0,1536 | 1,398 | 0,8441 | 1,3549 | 0,5087 |
|  | M467 | HET | SC | 0,1435 | 1,3268 | 0,8151 | 1,326 | 0,4577 |
|  | M471 | HET | SC | 0,0929 | 1,9065 | 0,7638 | 1,2139 | 0,4538 |
|  | M477 | HET | SC | 0,0804 | 2,2128 | 0,6887 | 1,3006 | 0,4781 |
|  | M432 | HET | MS | 0,1264 | 2,0918 | 0,6484 | 1,2345 | 0,3591 |
|  | M434 | HET | MS | 0,1241 | 1,5262 | 0,7328 | 1,2516 | 0,3781 |
|  | M458 | HET | MS | 0,0978 | 2,3797 | 0,6792 | 1,228 | 0,3653 |
|  | M459 | HET | MS | 0,0703 | 1,256 | 0,7333 | 1,2211 | 0,3824 |

Table descriptors: WT: wild-type, HET: *Mecp2*-heterozygous (*Mecp2*-het), MS: maternal separation, SC: standard care.

**# Supplemental Material: Statistical Analysis in R**

# 1. Load Required Packages

library(tidyverse) # Data manipulation

library(ARTool) # Aligned Rank Transformation ANOVA

library(car) # Levene’s test

library(rcompanion) # Post-hoc analysis

library(readxl) # Reading Excel files

# 2. Data Preprocessing

file_path <- "your_data_file.xlsx" # Update with actual file path

sheet_name <- "your_sheet_name" # Update with actual sheet name

df <- read_excel(file_path, sheet = sheet_name)

# Convert "-" to NA and ensure numerical variables are correctly formatted

numeric_vars <- c("Variable_1", "Variable_2", "Variable_3") # Replace with actual variable names

df[df == "-"] <- NA

df[numeric_vars] <- lapply(df[numeric_vars], as.numeric)

df <- na.omit(df)

# Convert categorical variables to factors

df$Genotype <- as.factor(df$Genotype)

df$Treatment <- as.factor(df$Treatment)

# 3. Normality and Homoscedasticity Tests

shapiro_results <- sapply(numeric_vars, function(var) shapiro.test(df[[var]])$p.value)

levene_results <- sapply(numeric_vars, function(var) {

leveneTest(df[[var]] ~ Genotype * Treatment, data = df)$"Pr(>F)"[1]

})

# Identify variable groups based on test results

anova_vars <- names(which(shapiro_results > 0.05 & levene_results > 0.05)) # Normal & homoscedastic

art_vars <- names(which(shapiro_results <= 0.05)) # Non-normal data

# 4. Statistical Analysis

## Two-way ANOVA for Normally Distributed Variables

anova_models <- lapply(anova_vars, function(var) {

anova_formula <- as.formula(paste(var, "~ Genotype * Treatment"))

aov(anova_formula, data = df)

})

names(anova_models) <- anova_vars

# Get ANOVA results

anova_results <- lapply(anova_models, summary)

anova_results

## Post-hoc Tests for Two-way ANOVA (Bonferroni Correction)

anova_posthoc <- lapply(anova_vars, function(var) {

pairwise.t.test(df[[var]], interaction(df$Genotype, df$Treatment), p.adjust.method = "bonferroni")

})

names(anova_posthoc) <- anova_vars

## ART ANOVA for Non-Normal Variables

art_models <- lapply(art_vars, function(var) {

art_formula <- as.formula(paste(var, "~ Genotype * Treatment"))

art(art_formula, data = df)

})

names(art_models) <- art_vars

# Get ART ANOVA results

art_anova_results <- lapply(art_models, anova)

art_anova_results

## Post-hoc Tests for ART ANOVA (Bonferroni Correction)

art_posthoc <- lapply(art_vars, function(var) {

pairwise.t.test(df[[var]], interaction(df$Genotype, df$Treatment), p.adjust.method = "bonferroni")

})

names(art_posthoc) <- art_vars

# 5. Summary of Statistical Tests Applied

cat("\nVariables analyzed with Two-way ANOVA:", anova_vars)

cat("\nVariables analyzed with ART ANOVA:", art_vars)

# For all tests, Bonferroni correction was applied to post-hoc comparisons.
